# *de novo MEPCE* nonsense variant associated with a neurodevelopmental disorder causes disintegration of 7SK snRNP and enhanced RNA polymerase II activation

**DOI:** 10.1038/s41598-019-49032-0

**Published:** 2019-08-29

**Authors:** Pauline E. Schneeberger, Tatjana Bierhals, Axel Neu, Maja Hempel, Kerstin Kutsche

**Affiliations:** 1 Institute of Human Genetics, University Medical Center Hamburg-Eppendorf, Hamburg, Germany; 2 Childrens Hospital, University Medical Center Hamburg-Eppendorf, Hamburg, Germany

**Keywords:** Genetics research, Molecular medicine

## Abstract

In eukaryotes, the elongation phase of transcription by RNA polymerase II (RNAP II) is regulated by the transcription elongation factor b (P-TEFb), composed of Cyclin-T1 and cyclin-dependent kinase 9. The release of RNAP II is mediated by phosphorylation through P-TEFb that in turn is under control by the inhibitory 7SK small nuclear ribonucleoprotein (snRNP) complex. The 7SK snRNP consists of the 7SK non-coding RNA and the proteins MEPCE, LARP7, and HEXIM1/2. Biallelic *LARP7* loss-of-function variants underlie Alazami syndrome characterized by growth retardation and intellectual disability. We report a boy with global developmental delay and seizures carrying the *de novo MEPCE* nonsense variant c.1552 C > T/p.(Arg518*). mRNA and protein analyses identified nonsense-mediated mRNA decay to underlie the decreased amount of MEPCE in patient fibroblasts followed by LARP7 and 7SK snRNA downregulation and HEXIM1 upregulation. Reduced binding of HEXIM1 to Cyclin-T1, hyperphosphorylation of the RNAP II C-terminal domain, and upregulated expression of *ID2*, *ID3*, *MRPL11* and snRNAs U1, U2 and U4 in patient cells are suggestive of enhanced activation of P-TEFb. Flavopiridol treatment and ectopic MEPCE protein expression in patient fibroblasts rescued increased expression of six RNAP II-sensitive genes and suggested a possible repressive effect of MEPCE on P-TEFb-dependent transcription of specific genes.

## Introduction

The regulation of gene transcription by RNA polymerase II (RNAP II) is crucial to developmental genes and those involved in stimulus-controlled pathways. RNAP II starts with transcription but pauses 30–60 nucleotides downstream of the transcription start site and needs additional stimuli to elongate along the length of the gene and terminate transcription after transcription end sites^1^. The release of RNAP II from pausing requires the positive transcription elongation factor b (P-TEFb), a heterodimeric cyclin-dependent kinase composed of the catalytic subunit cyclin-dependent kinase 9 (CDK9) and the regulatory component Cyclin-T1^2^. P-TEFb phosphorylates pause-inducing factors and serine residues within the carboxy-terminal heptapeptide repeat domain (CTD) of the largest RNAP II subunit to release RNAP II from the paused state^1^. Recruitment and activation of P-TEFb at particular gene promoters is a key regulatory step for productive elongation of transcription. P-TEFb itself is under stringent control as the majority of this kinase is sequestered in the nucleosol by the 7SK small nuclear RNP (7SK snRNP) complex composed of the non-coding 7SK snRNA and its interacting proteins LARP7, HEXIM1/2, and MEPCE^3^. LARP7 is a La-related protein family member and protects 7SK snRNA from degradation by binding its 3′ terminal end, HEXIM1 and HEXIM2 inhibit P-TEFb’s kinase activity, and MEPCE is the γ-methylphosphate capping enzyme of 7SK snRNA to enable its stability^2^. After binding 7SK snRNA, LARP7 and MEPCE interact and form the core snRNP^4^. HEXIM1/2 and P-TEFb subsequently assemble onto 7SK snRNP, ensuing inhibition of P-TEFb^5,6^. Release of P-TEFb from the 7SK snRNP complex is mediated by stress and specific posttranslational modifications, such as HEXIM1 phosphorylation, dephosphorylation and acetylation of CDK9 and Cyclin-T1, respectively, and MEPCE proteolysis^2,7^. Cleavage of MEPCE has been shown to concomitantly downregulate LARP7 protein amount and destabilize the core 7SK in megakaryocytes leading to release and activation of P-TEFb and enhanced phosphorylation of serine 2 (P-Ser2) in the CTD of the RNAP II^8^.

The 7SK snRNP complex has been recently linked to rare Mendelian disorders by the identification of homozygous loss-of-function mutations in *LARP7* in several members of a consanguineous family with facial dysmorphism, severe intellectual disability, and primordial dwarfism and in another consanguineous family with two individuals affected by intellectual disability^9,10^. The *LARP7*-associated phenotype, named Alazami syndrome, has been broadened by the description of a total of 18 individuals with biallelic *LARP7* mutations^9–15^. The core phenotype consists of postnatal growth retardation, severe intellectual disability and characteristic facial dysmorphism, including broad nose, malar hypoplasia, wide mouth, full lips and abnormally set teeth^14^.

Here we report a 5-year-old boy with global developmental delay, moderate intellectual disability, and intractable seizures. During the course of the disease, he showed progressive muscle weakness, a decline in physical capacities and cognitive abilities, and he developed an impulsive behavior with extreme tantrums and autistic features. By trio whole-exome sequencing we identified the *de novo* nonsense variant c.1552 C > T/p.(Arg518*) in *MEPCE* in the male individual. Our functional data indicate *MEPCE* haploinsufficiency as the most likely mechanism. The decrease in the amount of MEPCE protein to ~50% in patient fibroblasts was accompanied by simultaneous downregulation of LARP7 and 7SK snRNA, likely leading to a destabilized 7SK snRNP complex. Reduced binding of HEXIM1 to the P-TEFb component Cyclin-T1 and hyperphosphorylation of serine 2 in the CTD of the RNAP II in patient cells suggest a decreased portion of P-TEFb bound to the 7SK snRNP leading to ongoing P-TEFb activation. By comparing *MEPCE* haploinsufficent and *LARP7* knockout fibroblasts, we found similarities and differences in transcriptional regulation of some protein-coding and non-coding RNAP II-sensitive genes as well as 7SK snRNP-independent effects of MEPCE. Upregulated expression of specific RNAP II-dependent genes in *MEPCE* haploinsufficent patient cells could be rescued by inhibiting P-TEFb activity by flavopiridol and ectopic MEPCE expression and suggested a possible repressive MEPCE function on P-TEFb-dependent transcription of specific genes.

## Results

### Clinical data of the patient

The male patient is the second born child of a dizygotic male twin pair. The pregnancy was conceived by intracytoplasmic sperm injection. It was complicated by an exacerbation of the maternal inflammatory disease, requiring treatment with cortisone and beta-blockers. A mild enlargement of the cerebral ventricles was detected in the second twin. Fetal development was otherwise normal. The twins were delivered at 36 weeks of gestation via caesarean section. Birth measurements were within normal limits for both. The patient had a weight of 2360 g (−1.3 z), a length of 46 cm (−1.4 z) and an occipitofrontal head circumference (OFC) of 34 cm (0 z). Despite initial feeding difficulties, the patient thrived, and feeding improved within the following weeks. Brain ultrasonography during the first week of life confirmed a slight enlargement of the intraventricular spaces.

Since birth, the patient had generalized muscular hypertonia and displayed a reduced amount of spontaneous movements. Extensive physiotherapy did not improve his muscle tone. His motor development was delayed: he started to roll over at 11 months, to crawl at 16 months and to walk without support at 22 months. In addition, he showed poor coordination and impaired fine motor function skills. At the age of three years, muscular hypertonia evolved into progressive generalized muscular hypotonia. At the age of 4 years, he developed exercise-induced muscle pain and muscle weakness without features of destruction or degeneration of muscle tissue (rhabdomyolysis), leading to a markedly reduced walking distance. At the age of 5 years, he started to use a wheelchair for distances greater than 100 m.

Early speech development was delayed. At the age of 11 months, he started producing sounds after ear tube insertion into the eardrum to reduce recurring ear infections by accumulation of liquid in the middle ear cavity. Throughout the second and third year of life, he acquired a broad vocabulary and fluent speech, but with an indistinct pronunciation. After his third birthday, a progressive cognitive impairment became obvious: he lost comprehension skills and the ability to understand complex tasks and relations. His psychomotor development and cognitive, emotional and social skills were assessed by a standardized test (ET 6–6-R test) at the age of 4 years and 10 months that revealed deficits in social-emotional and cognitive development and fine motor skills. His behaviour became impulsive and aggressive and showed autistic characteristics.

At the age of 3 years, he displayed episodes of frequent eye and shoulder winks, misinterpreted as ticks first due to inconspicuous electroencephalograms (EEG). At the age of 4 ½ years, for the first time focal epilepsy-typical potentials (ETP) were detected in EEG, which correlated with winks and were more pronounced during sleep. Subsequently, epilepsy was diagnosed. Antiepileptic therapy reduced the frequency of winks, however, the winks did not disappear completely, and ETP’s poorly responded to therapy. Series of tonic seizures during sleep were first observed at the age of 5 years. Despite an enhancement of antiepileptic therapy, seizures could not be reduced permanently.

A first brain MRI at the age of 3 years and 9 months showed blurry frontal white matter lesions, prominent perivascular spaces in the frontal white matter and subcortical parietal and a hyperintense structure at the right os sphenoidale in T2-weighted images. These unspecific brain anomalies were stable in repeated brain MRIs at age of 4 years and 2 months and 5 years and 4 months.

**Figure 1:**
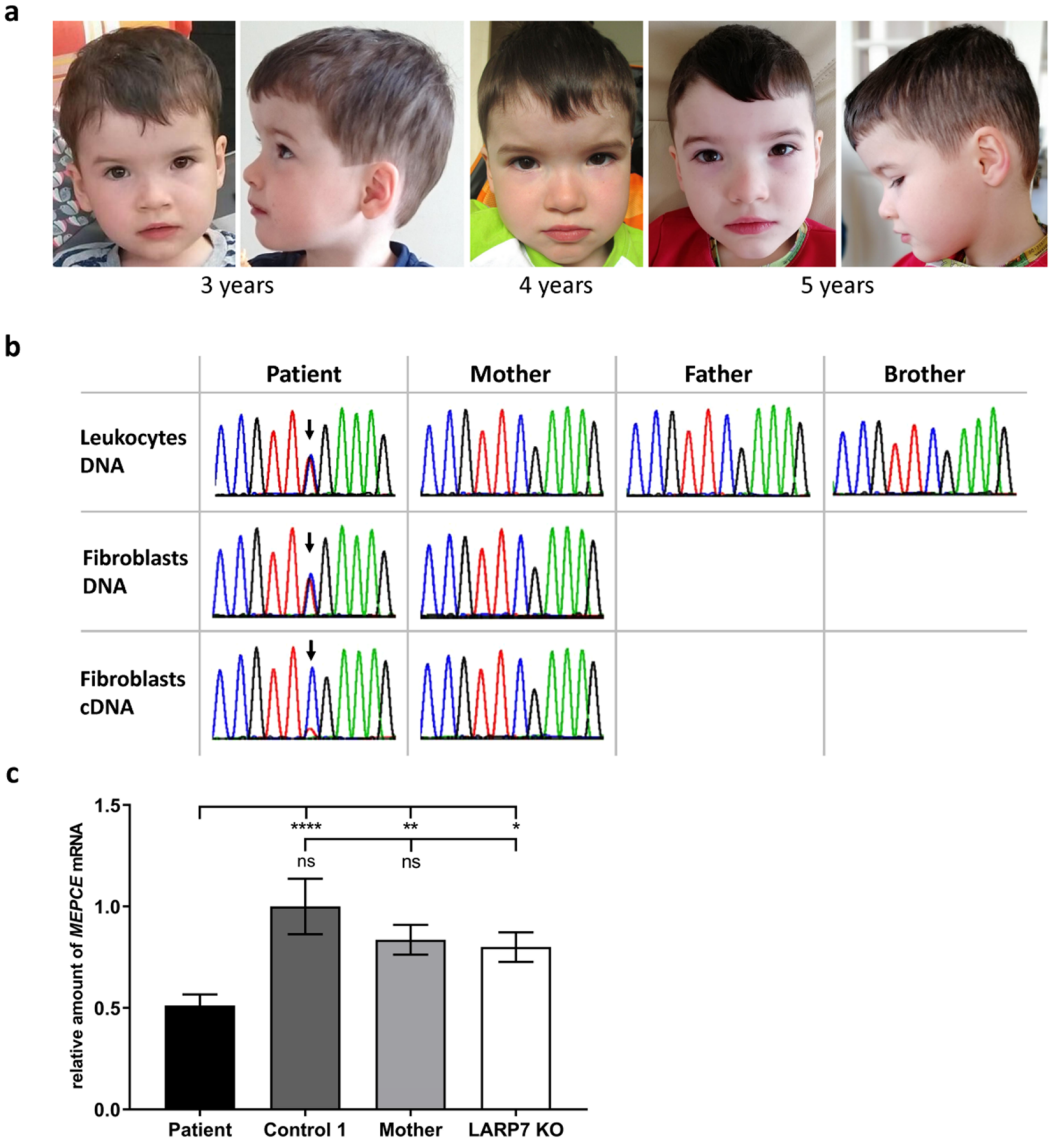
Photographs of the patient with the *de novo MEPCE* p.(Arg518*) variant and nonsense-mediated mRNA decay of mutated *MEPCE* transcripts in patient-derived fibroblasts. (**a**) Facial images of the patient at the indicated ages. Note prominent forehead, hypertelorism, deep-set eyes, broad nose, and flat and wide nasal bridge. (**b**) Partial sequence electropherograms show the *MEPCE* variant c.1552 C > T (indicated by an arrow) in leukocyte- and fibroblast-derived DNA of the patient. The variant is absent in leukocyte-derived DNA of both parents and his brother and also in fibroblast-derived DNA and cDNA of the patient’s mother. RT-PCR analysis showed predominant abundance of *MEPCE* wild-type transcripts in the patient’s fibroblasts suggesting nonsense-mediated mRNA decay of transcripts harboring the premature stop codon. (**c**) Quantification of *MEPCE* transcripts by RT-qPCR. RNA was obtained from fibroblasts of the patient, control 1, the patient’s mother and LARP7 KO. *GAPDH* mRNA was used as an internal control, and the amount of each analysed RNA relative to *GAPDH* mRNA is presented. The mean of four (patient and two healthy individuals) or three (LARP7 KO) independent experiments ± SD is given. *p ≤ 0.05; **p ≤ 0.01; ****p ≤ 0.0001 by one-way ANOVA followed by Bonferroni *post-hoc* test for multiple comparison. ns: not significant.

At his last examination at 5 years and 10 months, we saw a friendly and shy boy who was able to follow simple instructions and to answer simple questions appropriately. He showed mild facial dysmorphism with a prominent forehead and depressed nasal bridge (Fig. 1a). Examination revealed generalized muscular hypotonia in the presence of appropriate muscle strength. Deep tendon reflexes were diminished, in particular in the upper limbs. Interestingly, the upper limb tendon reflexes had always been weak and vanished at the age of 5 years. He had little muscle mass, particularly in his legs, suggesting muscular hypotrophy. His measurements were within the normal range with a length of 112.5 cm (−0.8 z), a weight of 18.5 kg (−0.9 z) and an OFC of 50 cm (−1.6 z). The family history was unremarkable with regard to neuro-(muscular) disease conditions but revealed an unspecified inflammatory disease in the twin sibling and the mother. Both had severe episodes of inflammation without focus, requiring anti-inflammatory treatment.

Repeated laboratory work-up, including extensive metabolic analyses, revealed normal results.

At the age of 5 years, somatosensory, visual and auditory evoked potentials were within normal limits, as well as nerve conduction velocity and electromyography. At the same age, eye examination, abdominal and kidney ultrasound, electro- and echocardiography revealed inconspicuous results.

### Identification of a *de novo MEPCE* nonsense mutation in the male patient with developmental delay, moderate intellectual disability, intractable seizures, and exercise intolerance

We performed trio or duo whole-exome sequencing (WES) in a total of 440 pediatric subjects with a neurodevelopmental disorder as described previously^16,17^. Analysis of WES data was performed according to X-linked, autosomal recessive, and autosomal-dominant inheritance models, the latter with a *de novo* mutation in the affected child. In the 5-year-old male patient with developmental delay, moderate intellectual disability and intractable seizures, we identified two *de novo* variants, the c.406 G > A variant in *MYO1G* (MIM: 600642), predicting the amino acid substitution p.(Asp136Asn), and the *MEPCE* (MIM: 611478) variant c.1552 C > T, predicting the introduction of a premature stop codon in the mRNA [p.(Arg518*)] (Table S1). Both variants were absent in dbSNP138, 1000 Genomes Project, Exome Variant Server, and ExAC and gnomAD browsers. Both genes have not yet been associated with rare Mendelian disorders, however, *MEPCE* was found to be overexpressed in highly tumorigenic breast cancer stem cells and promotes cellular invasion^18,19^. *De novo* occurrence of the *MEPCE* variant was confirmed by Sanger sequencing in leukocyte- and fibroblast-derived DNA of the patient (Fig. 1b). In addition, we detected the compound heterozygous variants c.445 + 4 A > G and c.17 G > A/p.(Arg6Gln) in the disease gene *TNFRSF13B* (MIM: 604907) [with minor allele frequency (MAF) < 0.1% in population databases (dbSNP138, 1000 Genomes Project, Exome Variant Server, ExAC and gnomAD browsers) and no homozygous carriers in the ExAC and gnomAD browsers] (Table S1). Heterozygous and biallelic variants in *TNFRSF13B* cause a form of common variable immunodeficiency (CVID2; MIM: 240500), characterized by hypogammaglobulinemia and recurrent infections, including otitis media, respiratory tract infections, and gastrointestinal tract infections^20,21^. The absence of CVID2-typical clinical features in the 5-year-old boy and consistent prediction of the two *TNFRSF13B* variants to be benign by various *in silico* pathogenicity and splice site prediction tools (Table S1) suggested the two variants not to be associated with the male patient’s neurodevelopmental phenotype. We next evaluated the two *de novo* variants in *MYO1G* and *MEPCE*. *In silico* tools consistently predicted the non-synonymous *MYO1G* variant c.406 G > A/p.(Asp136Asn) to be likely benign and to not affect pre-mRNA splicing (Table S1). This is in contrast to the *MEPCE* nonsense variant c.1552 C > T/p.(Arg518*) which has a CADD score of 35, predicting the variant to be damaging (Table S1)^22^. In addition, constraint metrices from ExAC and gnomAD for *MYO1G* indicated this gene to be tolerant to missense variants, while *MEPCE* is highly intolerant to loss-of-function variation, with a pLI score of 1 and a highly significant observed/expected score for protein-truncating variants of 0.04^23^. These data suggest that *MEPCE* belongs to the haploinsufficient class of genes and may represent a novel disease gene for a neurodevelopmental disorder.

### *MEPCE* haploinsufficiency likely underlies the developmental disorder in the patient

To analyse if the heterozygous *MEPCE* nonsense variant leads to nonsense-mediated mRNA decay (NMD)^24^ of *MEPCE* mutant transcripts, we isolated RNA from patient fibroblasts and performed qualitative RT-PCR followed by direct Sanger sequencing of the amplicon to detect wild-type and mutant *MEPCE* transcripts. As shown in the bottom panel of Fig. 1b, the peak for the mutant base thymine superimposed on the peak for cytosine was very small in fibroblast-derived cDNA of the patient. These data suggest that the amount of *MEPCE* transcripts with the mutant base thymine was drastically reduced in patient cells that is likely due to NMD of *MEPCE* mRNAs with the premature stop codon. To confirm this assumption, we performed RT-qPCR using RNA of patient-, patient’s mother- and control-derived fibroblasts as well as of fibroblasts derived from the published patient with the homozygous *LARP7* loss-of-function mutation c.1024_1030dup/p.(Thr344Lysfs*9) (LARP7 KO cells)^10^. We identified about half of the amount of *MEPCE* mRNA in the patient cells compared to LARP7 KO cells and cells of the two healthy individuals (Fig. 1c). Together, these data indicate that *MEPCE* transcripts expressed from the mutant allele are efficiently degraded by the NMD machinery resulting in a total amount of ~50% of *MEPCE* transcripts mainly expressed from the wild-type allele in patient cells.

**Figure 2:**
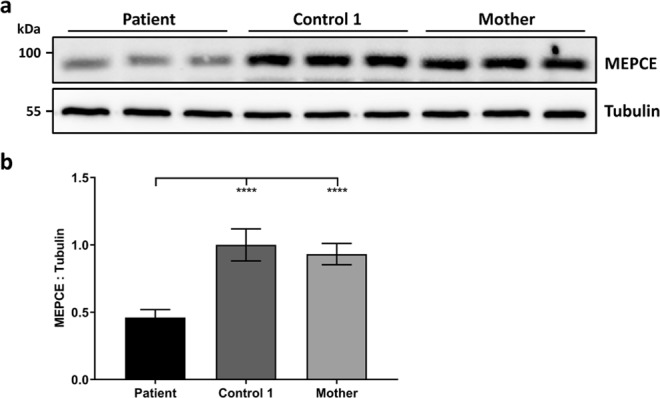
The heterozygous *MEPCE* nonsense variant leads to reduced amount of MEPCE protein in patient fibroblasts. (**a**) Immunoblot of lysates obtained from patient, control 1 and patient’s mother fibroblast cultures from three different passages. Endogenous MEPCE was monitored with an anti-MEPCE antibody, and anti-Tubulin antibody was used to control for equal loading. Representative blots are shown. Full-length blots are presented in Figure S1. (**b**) Band intensities were quantified using a chemiluminescence imager. MEPCE protein was normalized to Tubulin. The mean of six independent experiments ± SD is given. ****p ≤ 0.0001 by one-way ANOVA followed by Bonferroni *post-hoc* test for multiple comparison.

Next, we determined the MEPCE protein amount in fibroblasts of the patient, his mother and a control by immunoblotting and quantified band intensities. Similar to the *MEPCE* mRNA, MEPCE protein was reduced by ~54% in the cells of the patient compared to those of two control individuals (Figs 2a,b and S1). In addition, we did not observe a C-terminally truncated MEPCE mutant protein (p.Arg518*) with a theoretical molecular mass of ~55 kDa (Fig. S1), further underscoring the absence of *MEPCE* transcripts with the premature stop codon in patient fibroblasts. Together, *MEPCE* mRNA and protein analysis in the patient fibroblasts demonstrate a reduction of both to half of the amount compared to healthy individuals, suggesting that *MEPCE* haploinsufficiency likely is the pathomechanism rather than a dominant-negative effect of a putative MEPCE mutant protein lacking 172 C-terminal amino acid residues.

### The core components of the 7SK snRNP particle, such as 7SK snRNA and LARP7, are downregulated, and HEXIM1 is upregulated in patient cells

**Figure 3:**
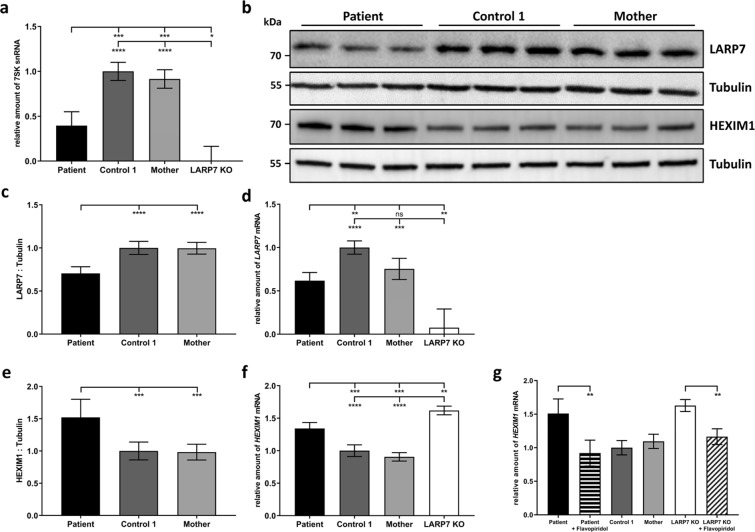
7SK snRNA and *LARP7* mRNA and protein amounts are decreased, while mRNA and protein levels of *HEXIM1* are increased in patient cells. (**a**) Quantification of the 7SK snRNA by RT-qPCR. *GAPDH* mRNA was used as an internal control, and the amount of each analysed RNA relative to *GAPDH* mRNA is presented. The mean of four (patient and two healthy individuals) or three (LARP7 KO) independent experiments ± SD is given. *p ≤ 0.05; ***p ≤ 0.001; ****p ≤ 0.0001 by one-way ANOVA followed by Bonferroni *post-hoc* test for multiple comparison. (**b**) Immunoblot of lysates obtained from patient, control 1 and patient’s mother fibroblast cultures from three different passages. Endogenous LARP7 and HEXIM1 were monitored with specific antibodies, and anti-Tubulin antibody was used to control for equal loading. Representative blots are shown. Full-length blots are presented in Figs S2 and S3. (**c**) Band intensities of LARP7 protein were quantified using a chemiluminescence imager, and LARP7 protein was normalized to Tubulin. The mean of six independent experiments ± SD is given. ****p ≤ 0.0001 by one-way ANOVA followed by Bonferroni *post-hoc* test for multiple comparison. (**d**) Quantification of *LARP7* transcript amount by RT-qPCR. *GAPDH* mRNA was used as an internal control, and the amount of each analysed RNA relative to *GAPDH* mRNA is presented. The mean of four (patient and two healthy individuals) or three (LARP7 KO) independent experiments ± SD is given. **p ≤ 0.01; ***p ≤ 0.001; ****p ≤ 0.0001 by one-way ANOVA followed by Bonferroni *post-hoc* test for multiple comparison. ns, not significant. (**e**) Band intensities of HEXIM1 protein were quantified using a chemiluminescence imager, and HEXIM1 protein was normalized to Tubulin. The mean of six independent experiments ± SD is given. ***p ≤ 0.001 by one-way ANOVA followed by Bonferroni *post-hoc* test for multiple comparison. (**f**) Quantification of *HEXIM1* transcript amount by RT-qPCR. *GAPDH* mRNA was used as an internal control, and the amount of each analysed RNA relative to *GAPDH* mRNA is presented. The mean of four (patient and two healthy individuals) or three (LARP7 KO) independent experiments ± SD is given. **p ≤ 0.01; ***p ≤ 0.001; ****p ≤ 0.0001 by one-way ANOVA followed by Bonferroni *post-hoc* test for multiple comparison. (**g**) Quantification of *HEXIM1* transcript amount by RT-qPCR after treatment of patient and LARP7 KO fibroblasts with 10 nM flavopiridol for 30 min. *GAPDH* mRNA was used as an internal control, and the amount of each analysed RNA relative to *GAPDH* mRNA is presented. The mean of three independent experiments ± SD is given. **p ≤ 0.01 by one-way ANOVA followed by Bonferroni *post-hoc* test for multiple comparison.

MEPCE is part of the canonical 7SK snRNP particle, consisting of 7SK snRNA and the LARP7 protein beside MEPCE^25,26^. Both LARP7 and MEPCE bind and stabilize the 7SK snRNA as depletion of either LARP7 or MEPCE via siRNA-mediated knockdown triggers 7SK snRNA degradation in cells^25,27–29^. In line with this, patient-derived LARP7 KO fibroblasts show complete lack of LARP7 protein and profound depletion of 7SK snRNA^10^. To analyse if the reduced MEPCE protein amount in patient cells has any effect on the core 7SK snRNP, we determined the relative amount of 7SK snRNA by RT-qPCR. We included cDNA from the published LARP7 KO fibroblasts^10^ in RT-qPCR experiments to compare the effects of *MEPCE* haploinsufficiency with those of complete LARP7 loss. We identified a profound reduction of the 7SK snRNA to ~40% in the patient fibroblasts compared to fibroblasts from the patient’s mother and a healthy control and confirmed absence of 7SK snRNA in LARP7 KO cells^10^ (Fig. 3a). Immunoblotting and quantification of band intensities revealed a significantly reduced amount of LARP7 protein by ~30% in patient compared to control cells (Figs 3b,c and S2). This downregulation of LARP7 possibly occurred at the transcriptional level as we found a similar reduction of the *LARP7* mRNA by ~38% in fibroblasts of the patient compared to control cells, while only ~7,6% of *LARP7* mRNA was left in LARP7 KO cells (Fig. 3d). HEXIM1, a central component of the 7SK snRNP, binds to and inhibits the kinase activity of P-TEFb^30–32^. Downregulation of the 7SK inhibitory scaffold causes upregulation of HEXIM1 through release of P-TEFb from the 7SK snRNP followed by increased expression of *HEXIM1*, a P-TEFb-dependent gene^33^. Thus, we next studied mRNA and protein levels of HEXIM1 and found an increase of both by 1.3- and 1.5-fold, respectively, in patient fibroblasts in comparison to the patient’s mother and control fibroblasts (Figs 3b,e,f and S3). Interestingly, LARP7 KO cells also showed a significantly elevated *HEXIM1* mRNA amount by 1.6-fold (Fig. 3f), suggesting that release of HEXIM1 proteins from the 7SK snRNP complex due to 7SK depletion may lead to activation of P-TEFb. To analyse if inhibition of P-TEFb activity by flavopiridol^34–36^ in patient and LARP7 KO cells leads to a decline in *HEXIM1* mRNA levels, we treated patient and LARP7 KO fibroblasts with flavopiridol and determined the relative amount of *HEXIM1* mRNA. We observed significantly reduced *HEXIM1* mRNA levels by ~39% and ~28% in flavopiridol-treated patient and LARP7 KO fibroblasts, respectively, compared to the respective untreated cells (Fig. 3g). These data suggest a P-TEFb-dependent increase in *HEXIM1* transcription in both *MEPCE* haploinsufficient and *LARP7* knockout fibroblasts suggesting higher P-TEFb activity in these cells. We also determined the protein level of the P-TEFb components CDK9 and Cyclin-T1 in patient- and two control-derived fibroblasts and did not observe any difference for the two proteins in the three investigated cell lines (Fig. S4). This data is in line with the observation of similar CDK9 protein levels in control and MEPCE-depleted breast cancer cells^37^. Taken together, *MEPCE* haploinsufficiency in the patient reported here as well as loss of LARP7 cause downregulation of the 7SK snRNP core components 7SK snRNA and LARP7, with subsequent transcriptional upregulation of *HEXIM1* in fibroblast cells. These findings suggest enhanced dissolution of the 7SK snRNP particle in patient and LARP7 KO cells that might be accompanied by release and activation of P-TEFb and subsequent transcriptional activation of P-TEFb target genes, such as *HEXIM1*.

### *MEPCE* haploinsufficiency leads to P-TEFb activation in fibroblasts of the patient

**Figure 4:**
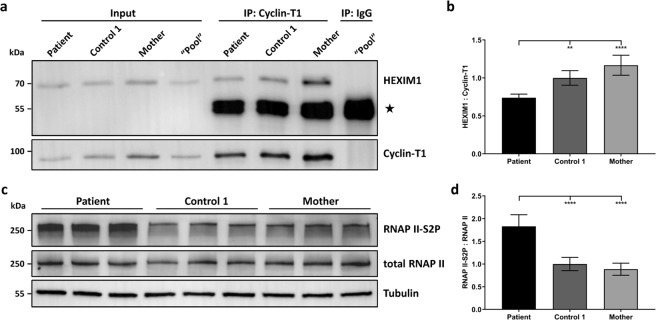
Binding of HEXIM1 to the P-TEFb component Cyclin-T1 is reduced, and the C-terminal domain of RNAP II is hyperphosphorylated in patient cells. (**a**) Endogenous Cyclin-T1 was immunoprecipitated from lysates obtained from patient-, control 1- and patient’s mother-derived fibroblasts. For control purposes, lysates from all three samples were pooled and incubated with an IgG isotype control antibody (“Pool”). Immunoprecipitates (IP) and total cell lysates (Input) were analysed by immunoblotting using the indicated antibodies. The band representing the antibody heavy chain is indicated with a star. Representative blots are shown. Full-length blots are presented in Fig. S5. (**b**) Band intensities were quantified with a chemiluminescence imager. The amount of co-immunoprecipitated HEXIM1 was normalized to the precipitated amount of Cyclin-T1. The mean of five independent experiments ± SD is given. **p ≤ 0.01, ****p ≤ 0.0001 by one-way ANOVA followed by Bonferroni *post-hoc* test for multiple comparison. (**c**) Immunoblot of lysates obtained from patient, control 1 and patient’s mother fibroblast cultures from three different passages. The amount of total and phosphorylated RNAP II (RNAP II-S2P) was monitored with specific antibodies, and anti-Tubulin antibody was used to control for equal loading. Representative blots are shown. Full-length blots are presented in Fig. S6. (**d**) Quantification of phosphorylated RNAP II. Band intensities were quantified with a chemiluminescence imager. The amount of RNAP II-S2P was normalized to total RNAP II. The mean of five independent experiments ± SD is given. ****p ≤ 0.0001 by one-way ANOVA followed by Bonferroni *post-hoc* test for multiple comparison.

To study if disintegration of the 7SK snRNP in patient cells leads to diminished interaction between the P-TEFb component Cyclin-T1 and HEXIM1, an interaction dependent on the integrity of the 7SK snRNP core complex^38^, we performed co-immunoprecipitation experiments. We immunoprecipitated endogenous Cyclin-T1 from cell lysates of the patient, the patient’s mother and one control and detected HEXIM1 in the immunoprecipitates. As shown in the representative immunoblot in Fig. 4a, the amount of co-precipitated HEXIM1 was reduced in patient compared to control 1 and patient’s mother cells (see also Fig. S5). Quantification of band intensities revealed a statistically significant reduction in the amount of co-immunoprecipitated HEXIM1 to ~74% in the patient compared to control 1, while the reduction was even more significant when compared to the mother (down to ~63% in the patient, Fig. 4b). The data indicate that MEPCE haploinsufficiency leads to reduced binding of HEXIM1 to P-TEFb that may be followed by a large-scale release of P-TEFb and its activation. To analyse if diminished sequestering of P-TEFb into the 7SK snRNP complex leads to increased P-TEFb activity, we studied the phosphorylation status of the CTD of RNAP II on serines at position 2 of its 52 heptapeptide (Y1-S2-P3-T4-S5-P6-S7) repeats (RNAP II-S2P) by using a specific antibody against RNAP II-S2P. P-TEFb’s kinase activity catalyzes the CTD phosphorylation that is required to stimulate transcription elongation^39,40^. Fibroblasts of the patient show a significant increase in the serine 2 phosphorylation status of the CTD of RNAP II (Figs 4c and S6). Quantification of the band intensities revealed a 1.8-fold increase in phosphorylation of RNAP II CTD serine 2 in the patient compared to control 1 cells and a 2.1-fold increase compared to the patient’s mother cells (Fig. 4d). Importantly, the increase in serine 2 phosphorylation of RNAP II was not accompanied by an increase in the total amount of RNAP II in the patient cells (compare total RNAP II with RNAP II-S2P in Fig. 4c). Together, our data suggest that patient cells have an enhanced amount of free P-TEFb leading to global P-TEFb activation and increased phosphorylation of serines at position 2 in the CTD heptapeptides of the RNAP II.

### Fibroblasts of the patient show upregulated expression of the protein-coding genes *ID2*, *ID3* and *MRPL11* and the major spliceosomal U1, U2 and U4 snRNAs that can be rescued by inhibition of P-TEFb activity and ectopic MEPCE expression

**Figure 5:**
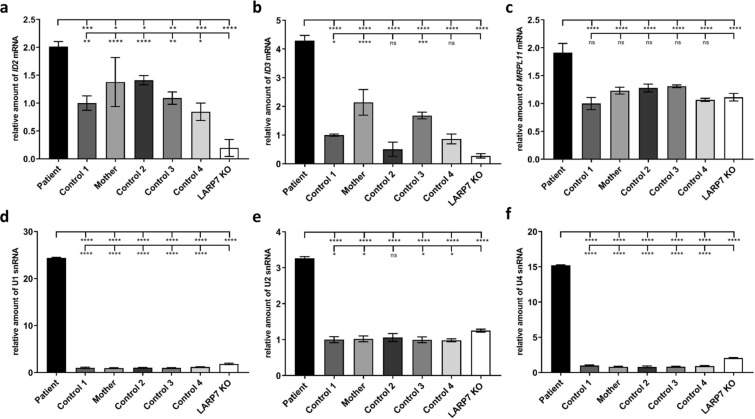
Expression of several RNAP II-synthesized genes is increased in patient-derived cells. Quantification of *ID2* (**a**), *ID3* (**b**) and *MRPL11* mRNAs (**c**) and U1 (**d**), U2 (**e**) and U4 snRNAs (**f** ) by RT-qPCR. RNA was obtained from fibroblasts of the patient, the patient’s mother, four healthy individuals (Control 1–4) and LARP7 KO cultured under normal conditions. *GAPDH* mRNA was used as an internal control, and the amount of each analysed RNA relative to *GAPDH* mRNA is presented. The mean of three independent experiments ± SD is given. *p ≤ 0.05; **p ≤ 0.01; ***p ≤ 0.001; ****p ≤ 0.0001 by one-way ANOVA followed by Bonferroni *post-hoc* test for multiple comparison. ns, not significant.

Upon release of P-TEFb from the 7SK snRNP complex, P-TEFb is recruited to RNAP II to facilitate productive elongation of transcription. P-TEFb primarily targets serine 2 of the CTD at actively transcribed genes and during transcription elongation^41,42^. In the breast cancer cell line MDA-MB-231, the genes *ID2* and *ID3* have been demonstrated to be under the control of P-TEFb, and reduced P-TEFb recruitment/activity has been suggested to underlie downregulated expression of *ID2* and *ID3* upon MEPCE depletion^37^. To study expression of the P-TEFb-regulated genes *ID2* and *ID3* in fibroblasts of the patient, we performed RT-qPCR using *ID2*- and *ID3*-specific primers. Interestingly, for both genes we could identify an increase in the amount of mRNA in the patient compared to four control and the patient’s mother cells (Fig. 5a,b). The *ID2* mRNA level was increased by 1.4- to 2.4-fold in patient fibroblasts compared to the cells of five healthy individuals (Fig. 5a). Although a variability in the *ID3* transcript amount was observed in the four control and the patient’s mother cells, patient cells showed a statistically significant enhanced amount of *ID3* mRNA by 2- to 8-fold when compared to each of the five control cell lines (Fig. 5b). While mRNA levels of *ID2* and *ID3* were increased in *MEPCE* haploinsufficient cells, mRNA levels of the two genes were drastically decreased in LARP7 KO cells: to 13.8–23.1% for *ID2* compared to all five control cells and to 12.8–27.5% for *ID3* compared to three of the five control cell lines (Fig. 5a,b). These data suggest different regulatory mechanisms to underlie dysregulated gene expression upon MEPCE depletion and LARP7 loss. Additional evidence for differences in transcriptional gene regulation in *MEPCE* haploinsufficient versus LARP7 KO cells came from RT-qPCR analysis of the randomly selected *MRPL11* gene: we identified a statistically significant increase in the *MRPL11* transcript level by 1.5- to 1.9-fold in patient compared to all control cells and LARP7 KO cells (Fig. 5c), suggesting a role of MEPCE but not LARP7 in transcriptional regulation of *MRPL11*. We also studied expression of *RIPK1*, a P-TEFb-regulated gene identified in immortalized human fibroblasts^43^. We did not detect any difference in the levels of *RIPK1* transcripts in patient and LARP7 KO cells compared to control cells (Fig. S7a). Similarly, no difference in mRNA levels was found for the randomly selected gene *TBC1D2B* (Fig. S7b).

MEPCE and LARP7 have been shown to bind other non-coding RNAs except 7SK. For example, both proteins bind the U6 major spliceosomal snRNA that is synthesized by RNA polymerase III^28,29,44–46^. We next asked the question whether the U6 snRNA level is altered in *MEPCE* haploinsufficient and LARP7 KO fibroblasts. RT-qPCR revealed no difference in the accumulation of U6 snRNA in the patient, LARP7 KO and five control fibroblast cell lines (Fig. S7c). This data is in line with the absence of any alteration in the U6 snRNA level upon depletion of *LARP7* or 7SK in HeLa cells^45,46^. In contrast, depletion of *LARP7* or 7SK in HeLa cells had drastic effects on accumulation of the RNAPII-synthesized major spliceosomal snRNAs U1, U2, and U4 and the ribosomal processing snoRNA U3 as the level of these four snRNAs was drastically reduced^45^. Based on this data Egloff *et al*. (2017) speculated that in patients with biallelic *LARP7* loss-of-function mutations the synthesis of spliceosomal snRNAs may be compromised leading to possible defects in pre-mRNA splicing^45^. We therefore analysed the level of the sn(o)RNAs U1, U2, U3 and U4 in LARP7 KO, MEPCE haploinsufficient and control fibroblasts. For the three major spliceosomal snRNAs U1, U2, and U4 we observed a slight increase in the RNA amount by 1.8-, 1.3-, and 2.1-fold, respectively, in LARP7 KO cells compared to control 1 cells (Fig. 5d–f), while accumulation of U1, U2, and U4 snRNAs was drastically increased in MEPCE haploinsufficent cells by 24.4-, 3.3-, and 15.2-fold, respectively, compared to control 1 cells (Fig. 5d–f). Interestingly, accumulation of the ribosomal processing U3 snoRNA was not affected in LARP7 KO and MEPCE haploinsufficient fibroblasts (Fig. S7d). Together, these data show that expression of some RNAP II-dependent genes, such as HEXIM1, is similarly dysregulated in fibroblasts with *MEPCE* haploinsufficiency and *LARP7* deficiency suggesting that the 7SK snRNP complex plays a regulatory role in expression of these genes. However, the differential effects on accumulation of *ID2*, *ID3* and *MRPL11* mRNAs and U1, U2 and U4 snRNAs in LARP7 KO and MEPCE haploinsufficient cell lines suggest differences in regulation of these RNAP II-transcribed genes in fibroblasts.

**Figure 6:**
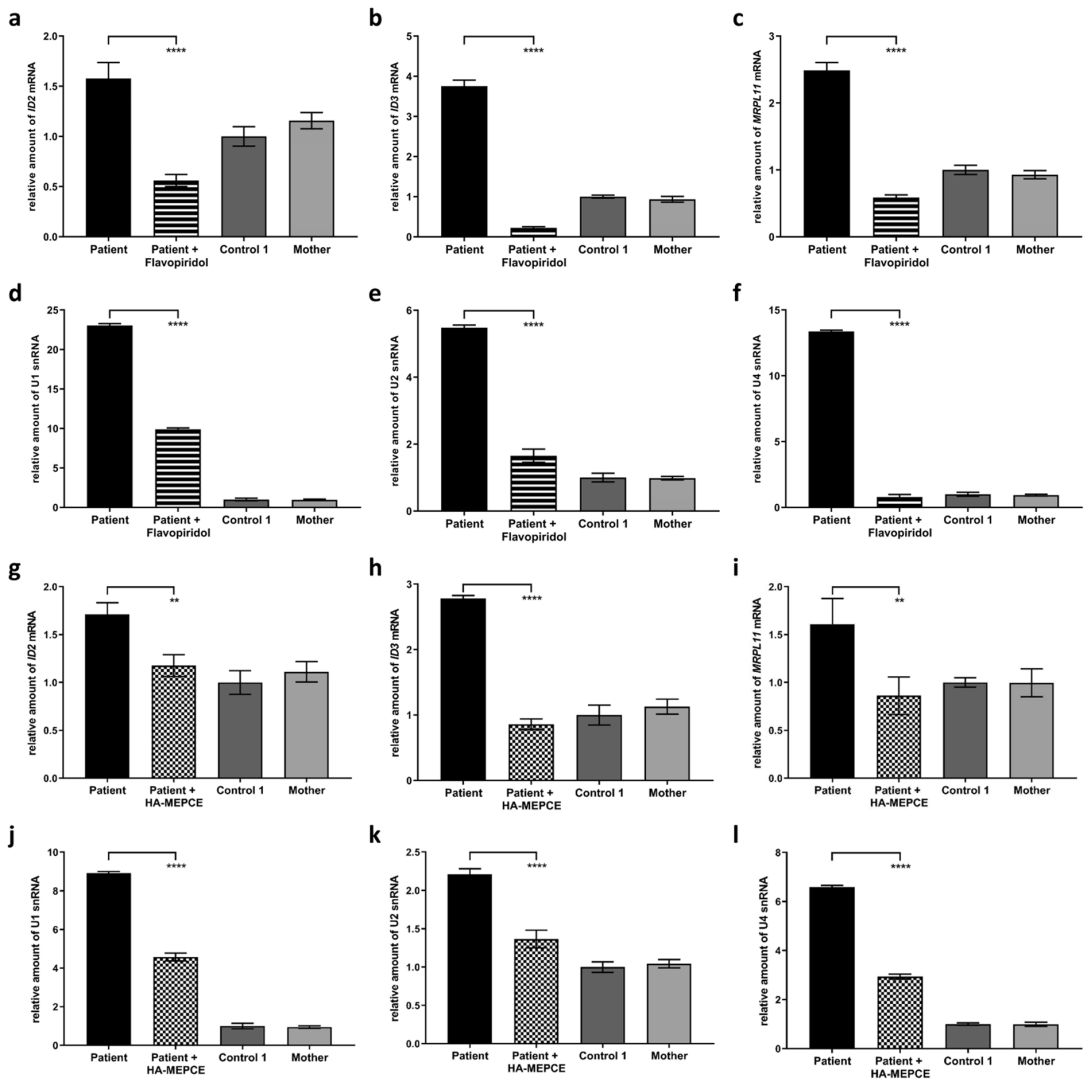
Upregulated expression of six RNAP II-dependent genes in patient fibroblasts can be rescued by treatment with flavopiridol or ectopic expression of HA-tagged MEPCE protein. Quantification of *ID2* (**a**,**g**), *ID3* (**b**,**h**), and *MRPL11* mRNAs (**c**,**i**), and U1 (**d**,**j**), U2 (**e**,**k**) and U4 **(f**,**l)** snRNAs by RT-qPCR. RNA was obtained from untreated fibroblasts of the patient, a healthy individual (control 1) and the patient’s mother as well as from patient cells treated with 10 nM flavopiridol (Patient + Flavopiridol) **(a–f)**. For data presented in **(g–l)**, fibroblasts of the patient were either transiently transfected with empty vector or HA-MEPCE expression construct (Patient + HA-MEPCE), while cells from a healthy individual (control 1) and the patient’s mother were only transfected with empty vector. *GAPDH* mRNA was used as an internal control, and the amount of each analysed RNA relative to *GAPDH* mRNA is presented. The mean of three independent experiments ± SD is given. **p ≤ 0.01; ****p ≤ 0.0001 by one-way ANOVA followed by Bonferroni *post-hoc* test for multiple comparison.

To analyse if the observed upregulated expression of several genes in *MEPCE* haploinsufficient cells respond to P-TEFb inhibition^34,35^, we treated patient cells with flavopiridol and studied accumulation of *ID2*, *ID3*, *MRPL11*, U1, U2 and U4 RNAs. Flavopiridol treatment caused a rapid and significant decline in the RNA level of all six analysed genes in treated compared to untreated patient cells (Fig. 6a–f). In another rescue experiment, we transiently transfected fibroblast cells of the patient with HA-tagged human MEPCE expression construct that yielded a transfection efficiency of 19.5% (Fig. S8a). Expression of HA-MEPCE protein in patient cells was confirmed by immunoblotting (Fig. S8b). The relative RNA level of *LARP7* and *HEXIM1* in HA-MEPCE expressing patient fibroblasts was comparable to control 1 and patient’s mother cells transfected with empty vector indicating successful rescue of *LARP7* and *HEXIM1* expression in patient cells (Fig. S9). However, 7SK snRNA amount only slightly increased in HA-MEPCE construct- versus empty vector-transfected patient cells (Fig. S9). RT-qPCR analysis to determine the relative RNA amount of *ID2*, *ID3*, *MRPL11*, U1, U2 and U4 revealed a significant decline in the level of the six transcripts in HA-MEPCE expressing patient fibroblasts compared to patient cells transfected with empty vector (Fig. 6g–l). Collectively, these data demonstrate a possible repressive function of MEPCE on transcription of several coding and non-coding RNAP II-synthesized genes that seems to be independent of the 7SK snRNP complex.

## Discussion

**Figure 7:**
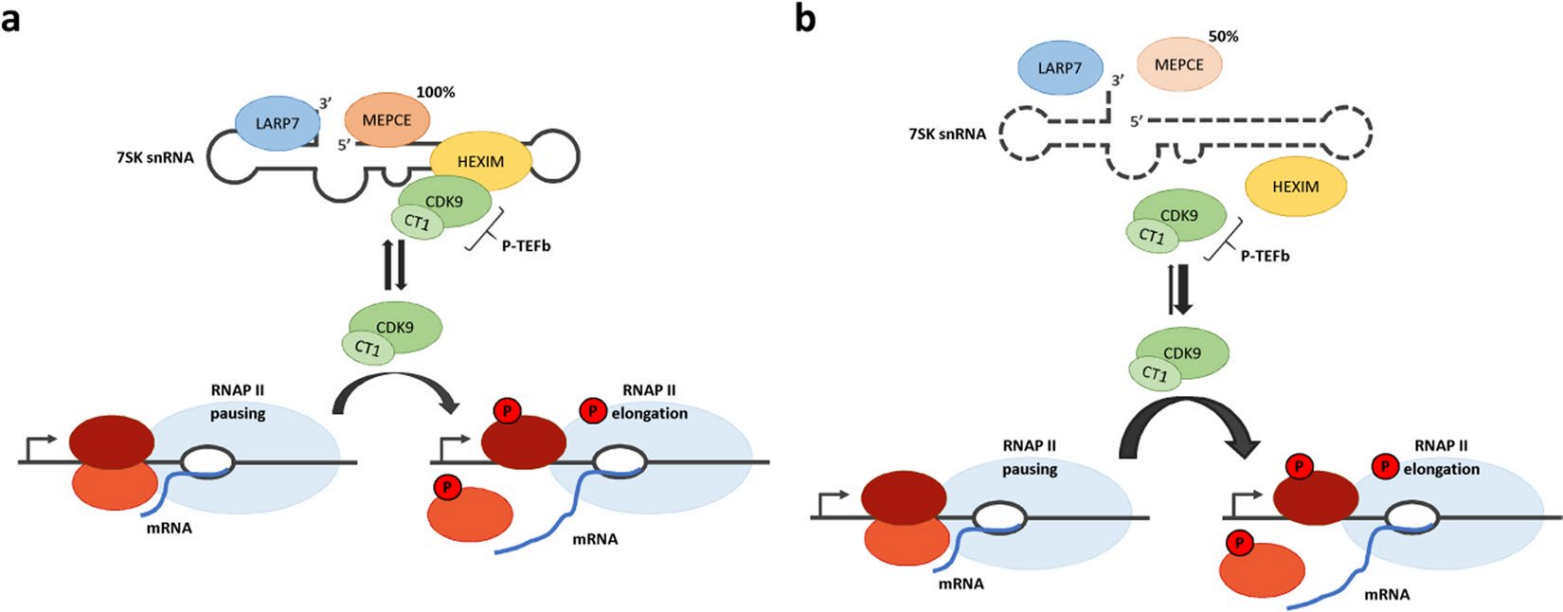
Model depicting the role of MEPCE in the 7SK snRNP complex and possible pathomechanism underlying the *MEPCE* nonsense variant. (**a**) In eukaryotes, synthesis of precursor mRNAs by RNA polymerase II (RNAP II) is essentially regulated by many factors (bottom figure). During the transcriptional process, RNAP II is paused proximal to the promoter by negative elongation factors (light and dark red ellipses). The release of RNAP II is mediated by phosphorylation through the positive transcription elongation factor b (P-TEFb) consisting of Cyclin-T1 (CT1) and the cyclin-dependent kinase 9 (CDK9). P-TEFb in turn is under control of the inhibitory 7SK small nuclear ribonucleoprotein (snRNP) complex (top figure). The 7SK snRNP core complex consists of the 7SK snRNA that is permanently bound to MEPCE and LARP7. MEPCE stabilizes the 7SK snRNA by 5′ cap methylation, and LARP7 protects the 7SK snRNA by binding its 3′ end. P-TEFb inhibition and incorporation in the 7SK snRNP complex is ensued by HEXIM1/2 dimers. Upon extra- and/or intracellular stimuli, P-TEFb is released from this complex and recruited to the paused RNAP II. Here, the CDK9 subunit of P-TEFb phosphorylates negative elongation factors which then dissociate from the complex or are inactivated. These steps are necessary for the release of paused RNAP II. In addition, P-TEFb phosphorylates specific serines in the C-terminus of RNAP II to stimulate the elongation of transcription. (**b**) The *MEPCE* nonsense mutation leads to a decrease in MEPCE protein amount by ~50% in patient-derived cells that is accompanied by depletion of 7SK snRNA and LARP7 protein. Consequently, disintegration of the 7SK snRNP complex likely leads to enhanced release and activation of P-TEFb, followed by hyperphosphorylation of RNAP II’s C-terminal domain. We postulate that the P-TEFb equilibrium is shifted toward free P-TEFb in patient cells leading to preferred transition of RNAP II from the paused to the productively elongating state and dysregulated expression of P-TEFb-regulated genes.

By analysis of the protein and mRNA amounts of components of the 7SK snRNP complex, the Cyclin-T1-HEXIM1 interaction, the phosphorylation status of the RNAP II CTD, and expression of RNAP II- and III- synthesized genes in fibroblasts of the patient with the *de novo MEPCE* nonsense mutation p.(Arg518*) and in LARP7 KO fibroblasts, we shed light on the molecular mechanisms underlying *MEPCE* haploinsufficiency and on similarities and differences in gene regulation upon *MEPCE* haploinsufficiency and LARP7 loss. We collectively demonstrate that *MEPCE* haploinsufficiency leads to disintegration of the 7SK snRNP complex accompanied by increased phosphorylation of RNAP II, possibly through enhanced release and activation of P-TEFb, that is followed by increased transcription of the protein-coding genes *HEXIM1*, *ID2*, *ID3* and *MRPL11* and the snRNAs U1, U2 and U4 (Model in Fig. 7). By inhibiting P-TEFb activity and ectopic MEPCE protein expression we could rescue upregulated expression of six RNAP II-sensitive genes in patient-derived fibroblasts suggesting a possible repressive effect of MEPCE on P-TEFb-dependent expression of these genes. Recently, it has been shown that the CDK9 kinase activity of P-TEFb can be inhibited by the transcriptional repressor CTIP2 (BCL11B)^47^, raising the possibility that MEPCE may control P-TEFb activity in a similar way.

Our data are in line with results obtained after knockdown of *MEPCE* in HeLa, HEK293 and breast cancer cell lines causing a decrease in the steady-state level of the 7SK snRNA, depletion of LARP7 protein, and diminished interaction between HEXIM1 and Cyclin-T1, concomitantly leading to a destabilized 7SK snRNP complex^25,28,37^. P-TEFb-dependent increase in *HEXIM1* transcription in both *MEPCE* haploinsufficient and *LARP7* knockout fibroblasts provides further evidence for disintegration of the 7SK snRNP complex and release of HEXIM1 from the complex. HEXIM1 can also bind to other non-coding RNAs, such as NEAT1, and serves as an RNA-dependent protein hub to form the HEXIM1-DNA-PK-paraspeckle RNP complex involved in innate immunity signaling^48^. We hypothesize that an excess in HEXIM1 protein amount followed by increased binding of HEXIM1 to NEAT1 may have consequences on DNA-mediated activation of innate immune response in the patient with the *MEPCE* nonsense mutation.

During megakaryopoiesis, downregulation of 7SK snRNP components is required to promote lineage-specific activation of P-TEFb and transcriptional upregulation of a set of genes encoding cytoskeletal remodeling factors, indicating an important role of the 7SK snRNP in specific developmental pathways^8^. This is further corroborated by the severe morphological defects observed upon knockdown of *Mepce* in zebrafish at 24 h postfertilization, including altered body axis formation and selective brain alterations suggestive of neurodegeneration. Interestingly, *Larp7* knockdown caused similar defects indicating that maintenance of the 7SK snRNP is essential for zebrafish development^25^. In *Drosophila*, Mepce (Bin3) is required for anterior-posterior pattern formation during embryogenesis^49^. Together, these data underscore the importance of the 7SK snRNP, including MEPCE and LARP7, for normal development in zebrafish and flies and also for neuronal development in humans (this work and^9–15^). However, while our data provide evidence of *MEPCE* haploinsufficiency being associated with developmental delay and moderate intellectual disability, *LARP7* needs to be knocked-out on both alleles to cause a neurodevelopmental disorder^10^. Accordingly, ExAC and gnomAD metrices indicate *LARP7* to be relatively tolerant to loss-of-function variation (with a pLI score of 0 and an observed/expected score for loss-of-function variants of 0.55), while *MEPCE* is highly intolerant to loss-of-function variation (see Results section). Our literature search did not reveal any putative pathogenic variant in *MEPCE* in whole-exome sequencing studies of large cohorts of individuals with developmental delay and/or intellectual disability^50–58^, suggesting that *de novo* truncating *MEPCE* variants are ultra rare in individuals with neurological anomalies. Moreover, the phenotype in subjects with biallelic *LARP7* variants is more severe than that in our patient as affected individuals show a spectrum of growth retardation ranging from short stature to primordial dwarfism associated with severe intellectual disability and distinct facial features^12,14^. Although *MEPCE* haploinsufficieny and *LARP7* knockout seem to affect 7SK snRNP stability in a similar way (see Fig. 3a,d,f and^10^), the consequences at the organismal levels may be different and depend on additional cellular functions of LARP7 and MEPCE as well as their interactome^18,28^. For example, both LARP7 and MEPCE, in the context of the 7SK snRNP complex, regulate expression of snRNAs and snoRNAs^45^ and stimulate pre-mRNA alternative splicing^25^. However, Mepce and 7SK snRNA can serve as scaffold for binding other proteins than Larp7, Hexim1 and P-TEFb, and this distinct 7SK snRNP is required for translation repression during *Drosophila* development^49,59^. In addition, MEPCE has 7SK snRNP-independent functions by binding the histone H4 tail and regulating P-TEFb activation on chromatin. Interestingly, the observed cellular effects of *MEPCE* depletion in this particular context were dominant over those of *LARP7* depletion suggesting that chromatin-bound MEPCE has a more important role in regulating P-TEFb activity than LARP7^37^. By comparing the transcript amount of several RNAP II-sensitive genes in *MEPCE* haploinsufficient and LARP7 KO cells we provide additional evidence for distinct functions of MEPCE and LARP7 in regulating expression of certain genes. While the *ID2*, *ID3* and *MRPL11* mRNA amount was significantly increased in *MEPCE* haploinsufficient cells, LARP7 KO cells showed a reduced transcript level of *ID2* and *ID3* and no change in *MRPL11* mRNA compared to control cells. In *MEPCE* haploinsufficient cells a drastic increase in accumulation of U1, U2 and U4 snRNAs was observed, whereas LARP7 KO cells showed only a slightly higher accumulation of the three snRNAs than control cells. Thus, *MEPCE* haploinsufficency could have 7SK snRNP-independent effects that may be related to MEPCE’s regulatory role in activating chromatin-bound P-TEFb^37^ or a not yet characterized repressive function of MEPCE in transcriptional regulation. Based on our and published data it is not surprising that the phenotypes associated with a heterozygous truncating *MEPCE* variant and biallelic *LARP7* loss-of-function variants are overlapping but vary in clinical manifestations and severity.

What could be the consequences of depleting the non-coding 7SK RNA and/or components of the 7SK snRNP complex on the nervous system? The 7SK snRNA and its associated proteins, such as CDK9, have been shown to be important for neuronal development. For example, the 7SK snRNA is strongly upregulated during neural differentiation and shows high expression levels in differentiating neurons. Knock-down of 7SK snRNA in embryonic stem cells compromised neuronal differentiation^60,61^. A function of the 7SK snRNA in axon elongation and maintenance of motor neurons has also been demonstrated as depletion of this non-coding RNA causes defective axon growth^62^. And Cdk9, one of the two components of P-TEFb, is required for survival of adult glial cells in the *Drosophila* brain^63^. Thus, these data demonstrate the importance of the 7SK snRNP particle during neuronal development and for normal brain function. Moreover, the *ID* gene family, which shows upregulated expression in patient cells with the heterozygous *MEPCE* null allele, encode helix-loop-helix (HLH) transcription factors that cannot bind to DNA. ID proteins act in a dominant-negative manner by forming heterodimers with other DNA-binding members of the HLH family and disrupting the protein-DNA interaction^64^. *ID* mRNA expression is high during growth and development^65^. The encoded proteins critically determine the cell’s eventual fate^66,67^ and are important for cellular differentiation and proliferation in the mammalian nervous system^68–70^. An interesting link between altered *ID* gene expression and intellectual disability has been reported: the genes *ID1*, *ID2*, *ID3*, and *ID4* are neuronal targets of MECP2, a transcriptional repressor, mutated in the X-linked neurodevelopmental disorder Rett syndrome. In *Mecp2*-deficient mouse brain significantly increased levels of all ID proteins have been observed that may contribute to the molecular pathogenesis in patients with Rett syndrome^71^. As Id1, Id2, and Id3 increase the proliferation potential of neural stem cells and inhibit neuronal differentiation by regulating activity of the basic HLH transcription factor HES1 in mice^69,72,73^, possible upregulated expression of these genes in the brain of individuals with a heterozygous *MEPCE* loss-of-function mutation may affect neurogenesis and neuronal differentiation.

In conclusion, by our in-depth functional studies, we demonstrate that *MEPCE* haploinsufficiency is the likely cause in the patient with intellectual disability, exercise intolerance and intractable seizures. Depletion of the 7SK snRNA and associated proteins seems to be the common pathomechanism underlying the neurodevelopmental phenotypes associated with a heterozygous *MEPCE* null allele and biallelic *LARP7* loss-of-function mutations and highlights the importance of the 7SK snRNP complex in human neurodevelopment. However, 7SK snRNP-independent functions of MEPCE and LARP7 likely account for clinical differences in *MEPCE*- and *LARP7*-related disorders. For example, MEPCE seems to have additional targets on RNAP II-synthesized transcripts and may mediate negative regulation of P-TEFb activity in the context of yet to be defined protein complexes. The recently identified homozygous *CDK9* missense mutation p.(Arg225Cys) in three families with individuals affected by developmental delay, seizures, choanal atresia, eye coloboma or cataract and visual impairment as common features emphasizes P-TEFb to also play a role in human development^74,75^.

## Material and Methods

### Study approval

The study was approved by the Ethics Committee of the Hamburg Medical Chamber (reference number PV3802) and undertaken with prior informed consent. Informed consent for skin biopsy and publication of photographs was obtained from both parents of the patient and the patient’s mother. All experiments were performed in accordance with relevant guidelines and regulations.

### Exome sequencing, sequence data analysis, and variant validation

Genomic DNA was extracted from peripheral blood samples using standard procedures. We performed trio whole-exome sequencing (trio WES) with DNA samples of the patient and both healthy parents as described before^16,17^. Briefly, coding DNA fragments were enriched with a SureSelect Human All Exon 50 Mb V5 Kit (Agilent), and captured libraries were then loaded on a HiSeq2500 platform (Illumina). Reads were aligned to the human reference genome (UCSC GRCh37/hg19) using the Burrows-Wheeler Aligner (BWA, v.0.5.87.5), and detection of genetic variation was performed with SAMtools (v.0.1.18), PINDEL (v. 0.2.4t), and ExomeDepth (v.1.0.0). Approximately 99% of target sequences were covered at least 20-fold with a mean coverage of at least 214×. Exonic and splice variants were prioritized by pathogenicity assessment using multiple *in silico* tools (CADD, REVEL, M-CAP, Human Splicing Finder 3.1, NetGene2-Server, and Berkeley Drosophila Genome Project-Database).

*MEPCE* (mRNA reference sequence: NM_019606.6) variant validation in the family was performed by Sanger sequencing. Primer sequences are listed in Table S2. Amplicons were directly sequenced using the ABI BigDye Terminator Sequencing kit (Applied Biosystems) and an automated capillary sequencer (ABI 3500, Applied Biosystems). Sequence electropherograms were analysed using the Sequence Pilot software (JSI Medical Systems) and Chromas Lite 2.1.1 (Technelysium Pty Ltd). The variant details have been submitted to LOVD (https://databases.lovd.nl/shared/genes/MEPCE).

### Expression construct

pcDNA5/FRT/TO-Intron-CterFlag-MePCE was a gift from Blerta Xhemalce (Addgene plasmid #113549)^37^ and used to amplify the coding region of human *MEPCE* (NM_019606.6) using forward primer 5′-GCCGCGGTACCTCGAGCAATCGAGATGGCGGCGG-3′ and reverse primer 5′-CCCCCCCCCCGAATTCTTAGTGGCTGGGGGATCGG-3′. Relevant restriction sites in primer sequences (*Xho*I in the forward and *Eco*RI in the reverse primer) allowed for recombination of the amplicon in pMT2SM-HA vector by In-Fusion HD Cloning (TaKaRa) according to the manufacturer’s protocol. All constructs were sequenced for integrity.

### Cell culture

Primary fibroblasts obtained from a skin biopsy of the patient, his mother and one healthy control individual were cultured in Dulbecco’s modified Eagle medium (DMEM; Thermo Fisher Scientific) supplemented with 10% fetal bovine serum (FBS; GE Healthcare) and penicillin-streptomycin (100 U/mL and 100 mg/mL, respectively; Thermo Fisher Scientific). Patient-derived fibroblasts with the homozygous *LARP7* mutation c.1024_1030dup/p.(Thr344Lysfs*9) (NM_016648.4; LARP7 KO cells) were a kind gift from Fowzan S. Alkuraya^10^. Integrity of the cell line was assessed by Sanger-sequencing of genomic DNA revealing the presence of the *LARP7* mutation. Cells were tested for mycoplasma contamination and confirmed to be mycoplasma free.

For inhibition of CDK9, fibroblasts were seeded in a T25 flask and incubated under normal culture condition overnight. Next day, cells were treated with either 10 nM flavopiridol (Sigma-Aldrich) in 0.9% NaCl for 30 min or left untreated. Then, cells were trypsinized and RNA was isolated.

For ectopic HA-tagged MEPCE expression, fibroblasts were seeded in 6-well plates and transiently transfected with either HA-MEPCE-pMT2SM-HA expression construct or empty pMT2SM-HA vector using VIROMER RED (Lipocalyx) according to manufacturer’s instructions. After 24 h, cells were harvested for RNA isolation or protein analysis.

### RNA isolation, cDNA synthesis and reverse transcription (RT) quantitative PCR (RT-qPCR)

Total RNA was extracted (RNeasy Mini Kit, Qiagen) from cultured primary fibroblasts of the patient, his mother and healthy individuals. RNA concentration and purity of the samples were assessed by use of the Microplate Spectrophotometer Epoch (BioTek). 1 µg total RNA was reverse transcribed (Superscript III RT, Thermo Fisher Scientific) using random hexamers.

Technical duplicates of RT-qPCR samples were prepared as a 10 µL approach with the SYBR Green I-based Luna Universal qPCR Master Mix (New England BioLabs), 500 nM of each primer, and 1 µl of the reverse transcription reaction (cDNA). Primer sequences are listed in Table S2. RT-qPCR was performed using the QuantStudio 3 Real-Time PCR System (Thermo Fisher Scientific) equipped with QuantStudio Design&Analysis Software v1.4.3 (Thermo Fisher Scientific). The PCR conditions included a pre-run at 95 °C for 5 min, followed by 40 cycles of 30 s at 95 °C, 30 s at 58 °C and 45 s at 72 °C. PCR amplification specificity was determined by melting curve analysis with a range from 60 °C to 95 °C. The values of the cycle threshold (CT) of the target genes were normalized to the housekeeping gene *GAPDH*. For relative gene expression the comparative cycle threshold (ΔΔCT) values were calculated with the QuantStudio Design&Analysis Software (Thermo Fisher Scientific) with *GAPDH* as internal control and expressed as x-fold change to control 1.

### Antibodies

The following primary antibodies and dilutions were used: rabbit monoclonal anti-CDK9 (1:1,000, Cell Signaling Technology [CST], #2316); rabbit monoclonal anti-Cyclin-T1 (WB: 1:1,000, IP: 1:100, CST, #81464); mouse monoclonal anti-HA.11 Epitope Tag (1:500, Covance, #901501); rat monoclonal anti-HA HRP-coupled antibody (1:5,000, Roche, #12013819001); rabbit monoclonal anti-HEXIM1 (1:1,000, CST, #12604); rabbit polyclonal anti-LARP7 (1:1,000, Proteintech, #17067-1-AP); rabbit polyclonal anti-MEPCE (1:1,000, Proteintech, #14917-1-AP); rabbit polyclonal anti-RNA Polymerase II (1:1,000, Bethyl Laboratories, #A300-653A); rabbit polyclonal anti-phospho RNA polymerase II (S2) (1:1,000, Bethyl Laboratories, #A300-654A); mouse monoclonal anti-Tubulin antibody (1:5,000, Sigma-Aldrich, #T9026).

The following secondary antibodies and dilutions were used: sheep anti-mouse IgG HRP-coupled antibody (1:10,000, GE Healthcare, #NA931V); donkey anti-rabbit IgG HRP-coupled F(ab’)_2_ fragment (1:5,000, GE Healthcare, #NA9340V); goat anti-mouse IgG Alexa Fluor 488-coupled antibody (1:1,000, Invitrogen, #A-11001).

### Immunoblotting

Patient and control fibroblasts were harvested in ice-cold RIPA buffer (50 mM Tris-HCl pH 8.0, 150 mM NaCl, 1% NP-40, 0.5% DOC, and 0.1% SDS) supplemented with Mini Protease Inhibitor and PhosSTOP (Roche) and lysed for 10 min on ice. Cell debris was removed by centrifugation for 10 min at 4 °C, and 4x sample buffer (33% glycerol, 80 mM Tris-HCl pH 6.8, 0.3 M DTT, 6.7% SDS, 0.1% bromophenol blue) was added to the supernatants. Protein extracts were separated on SDS-PAGE under denaturing conditions and transferred to PVDF (polyvinylidene fluoride) membranes. Membranes were blocked followed by incubation with the indicated primary antibody at 4 °C overnight and with HRP (horseradish peroxidase)-linked secondary antibodies at room temperature (RT) for 1 h. Chemiluminescent immunoblots were digitally imaged with a ChemiDoc MP (Bio-Rad). Band intensities were determined with the Image Lab v6.0 software (Bio-Rad).

### Co-immunoprecipitation

Patient, patient’s mother and control fibroblasts were lysed in 500 µL ice-cold co-immunoprecipitation buffer (120 mM NaCl, 50 mM Tris-HCl pH 8.0, 0.5% NP-40, 1 mM EDTA), supplemented with complete Mini Protease Inhibitors (Roche), and cell lysates were clarified by centrifugation for 10 min at 4 °C. After removing an aliquot (Input), supernatants were supplemented with the indicated primary antibody, transferred to 40 µL Protein A-Agarose beads (Roche) and incubated for 4 h at 4 °C under rotating conditions. For control purposes, lysates from the three samples were pooled and incubated with Normal Rabbit IgG Polyclonal Antibody (1 µg; EMD Millipore, #12–370). Precipitates were collected by four times centrifugation and washing with 500 µL cold co-immunoprecipitation buffer (2500 g, 4 °C, 2 min). After final washing, beads were resuspended in 25 µL 4x sample buffer, and supernatant was subjected to SDS-PAGE and immunoblotting.

### Immunocytochemistry

Patient fibroblasts were seeded on glass coverslips in 6-well plates and transiently transfected as described above. 24 h after transfection, cells were fixed with 4% paraformaldehyde (Sigma-Aldrich) in PBS. After treatment with permeabilization/blocking solution (2% BSA, 3% goat serum, 0.5% Nonidet P40 in PBS), cells were incubated in antibody solution (3% goat serum, 0.1% Nonidet P40 in PBS) containing primary antibody for 3 h at room temperature. Cells were washed with PBS and incubated with secondary antibody in antibody solution for 1 h at room temperature. After extensive washing with PBS, cells were embedded in mounting solution (ProLong Diamond Antifade Mountant with DAPI; Thermo Fisher Scientific). Cells were analysed with Zeiss Axiovert 200 M epifluorescence microscope equipped with a 20x LD Plan-Neofluar Korr Ph2 objective lens, and images were captured and processed by AxioVision 4.8.2 software.

### Data analysis and statistics

The ExpressionSuite Software v1.1 (Thermo Fisher Scientific) was used for analysis of multiple RT-qPCR data sets (biological replicates). Quantitative data are presented by GraphPad prism8 software (Instat, GraphPad Software) as the mean ± standard deviation (SD). For all experiments, statistical analysis was performed via one-way analysis of variance (ANOVA) followed by a Bonferroni *post-hoc* test for multiple comparisons. A *p* value of less than 0.05 was considered statistically significant (*p ≤ 0.05; **p ≤ 0.01; ***p ≤ 0.001; ****p ≤ 0.0001).

## Supplementary information

Dataset 1

## Data Availability

All data generated or analysed during this study are included in this published article (and its Supplementary Information file).
