# Supplementary material for: de novo MEPCE nonsense variant associated with a neurodevelopmental disorder causes disintegration of 7SK snRNP and enhanced RNA polymerase II activation
Source: Sci Rep. 2019 Aug 29;9:12516. doi: 10.1038/s41598-019-49032-0 (PMC6715695; doi:10.1038/s41598-019-49032-0)
Supplement: Supplementary file 1 — Dataset 1 [file 41598_2019_49032_MOESM1_ESM.pdf]

***de novo MEPCE* nonsense variant associated with a neurodevelopmental disorder causes disintegration of 7SK snRNP and enhanced RNA polymerase II activation**

Pauline E. Schneeberger, Tatjana Bierhals, Axel Neu, Maja Hempel, Kerstin Kutsche

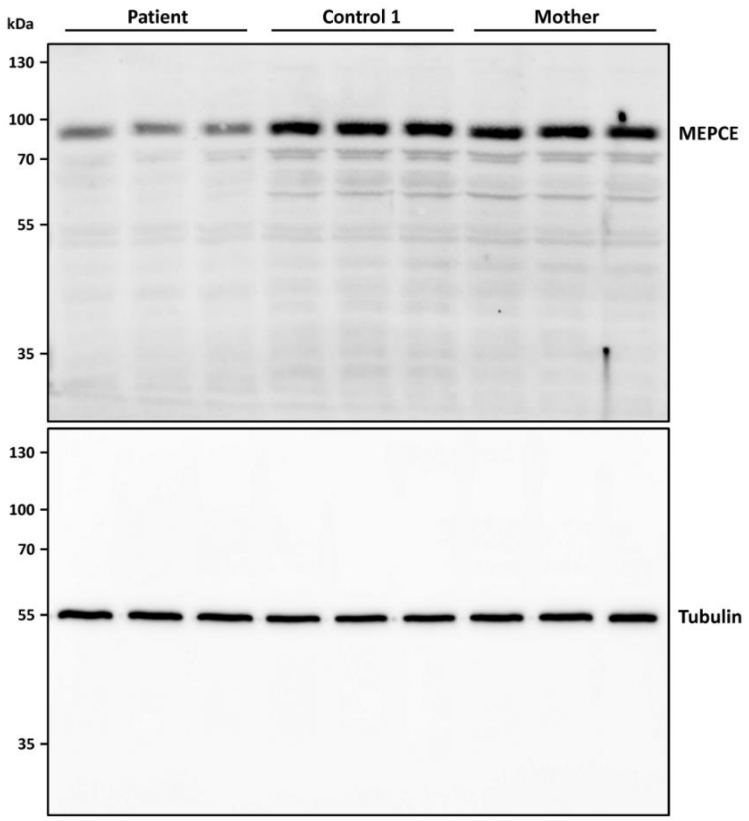

Figure S1. Full-length blots of Figure 2a

Immunoblots of lysates obtained from patient, control 1 and patient's mother fibroblast cultures from three different passages. The amount of MEPCE was monitored with a specific antibody. An anti-Tubulin antibody was used to control for equal loading.

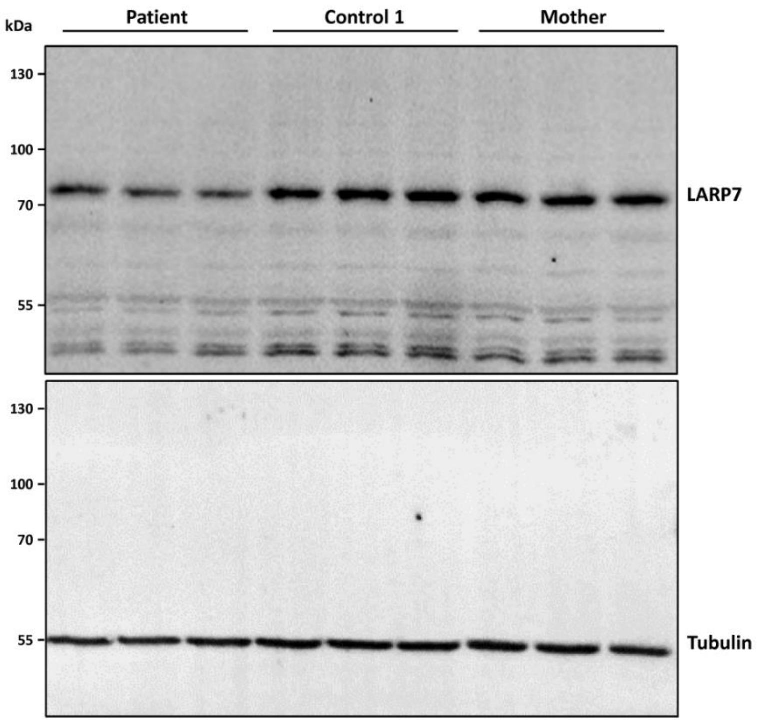

Figure S2. Full-length blots of Figure 3b (for LARP7)
Immunoblots of lysates obtained from patient, control 1 and patient's mother fibroblast cultures from three different passages. The amount of LARP7 was monitored with a specific antibody. An anti-Tubulin antibody was used to control for equal loading.

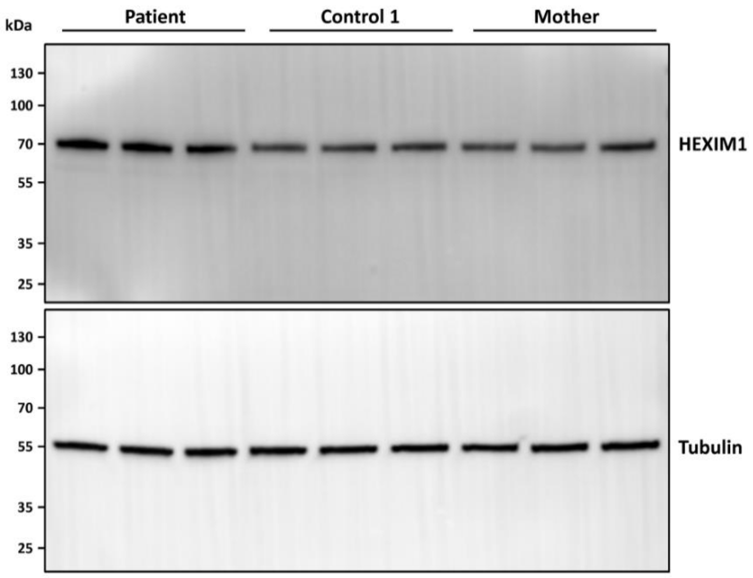

Figure S3. Full-length blots of Figure 3b (for HEXIM1)

Immunoblots of lysates obtained from patient, control 1 and patient's mother fibroblast cultures from three different passages. The amount of HEXIM1 was monitored with a specific antibody. An anti-Tubulin antibody was used to control for equal loading.

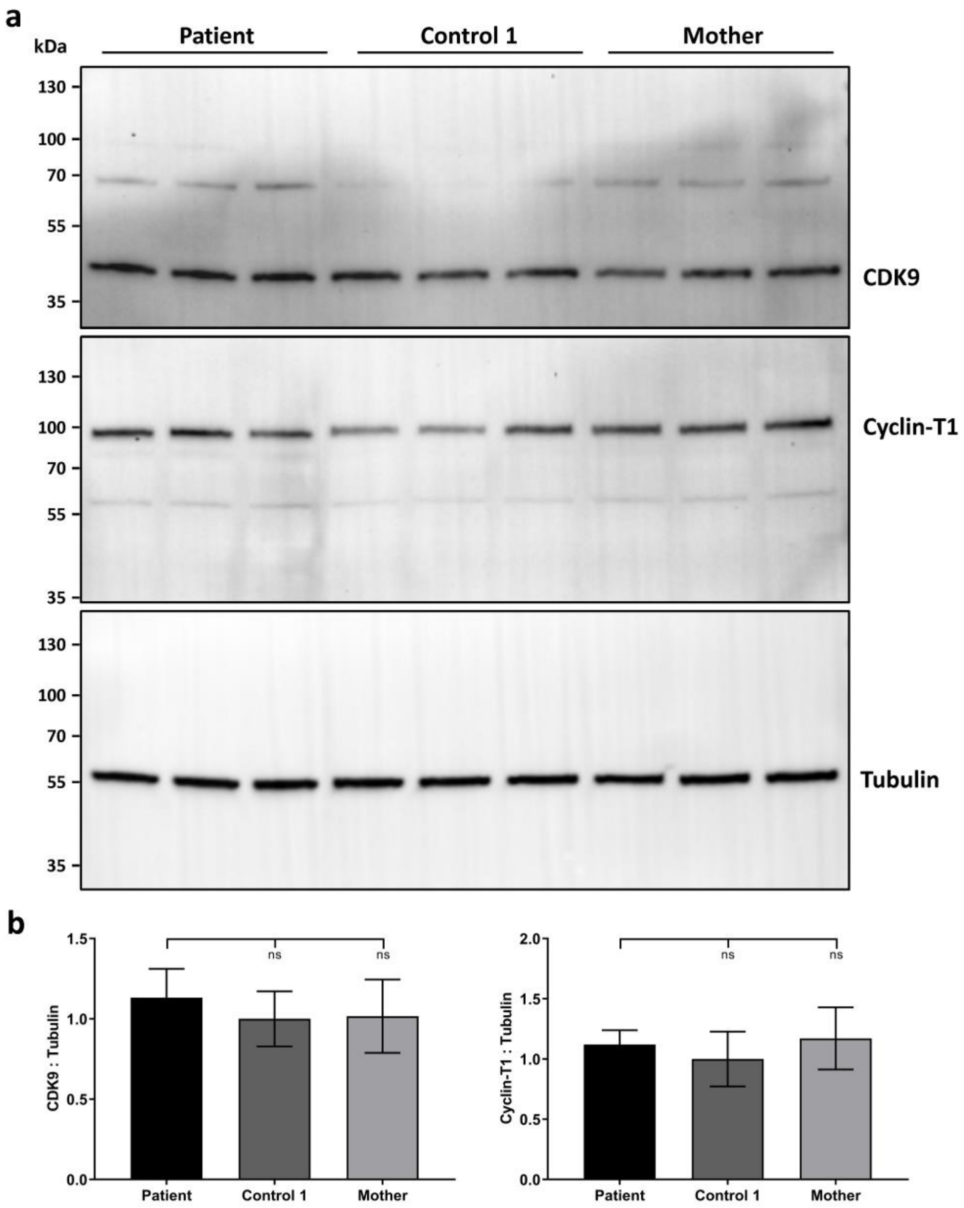

Figure S4. Protein amount of CDK9 and Cyclin-T1 is not altered in patient cells
(a) Full-length immunoblots of lysates obtained from patient, control 1 and patient's mother fibroblast cultures from three different passages. The amount of CDK9 and Cyclin-T1 was monitored with specific antibodies. An anti-Tubulin antibody was used to control for equal loading. Representative blots are shown. **(b)** Band intensities were quantified using a chemiluminescence imager. Protein amount of CDK9 (left panel) and Cyclin-T1 (right panel) was normalized to Tubulin. The mean of six independent experiments ± SD is given. A one-way ANOVA followed by Bonferroni *post-hoc* test for multiple comparison was performed. ns, not significant.

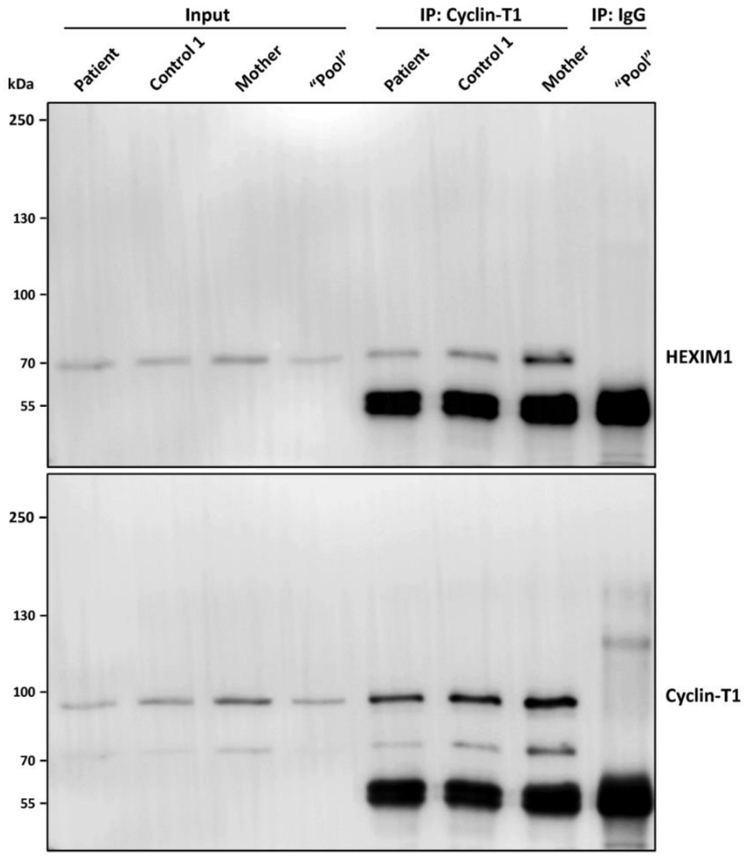

Figure S5. Full-length blots of Figure 4a

Endogenous Cyclin-T1 was immunoprecipitated from lysates obtained from patient-, control 1- and patient's mother-derived fibroblasts. For control purposes, lysates from all three samples were pooled and incubated with an IgG isotype control antibody ("Pool"). Immunoprecipitates (IP) and total cell lysates (Input) were analysed by immunoblotting using the indicated antibodies.

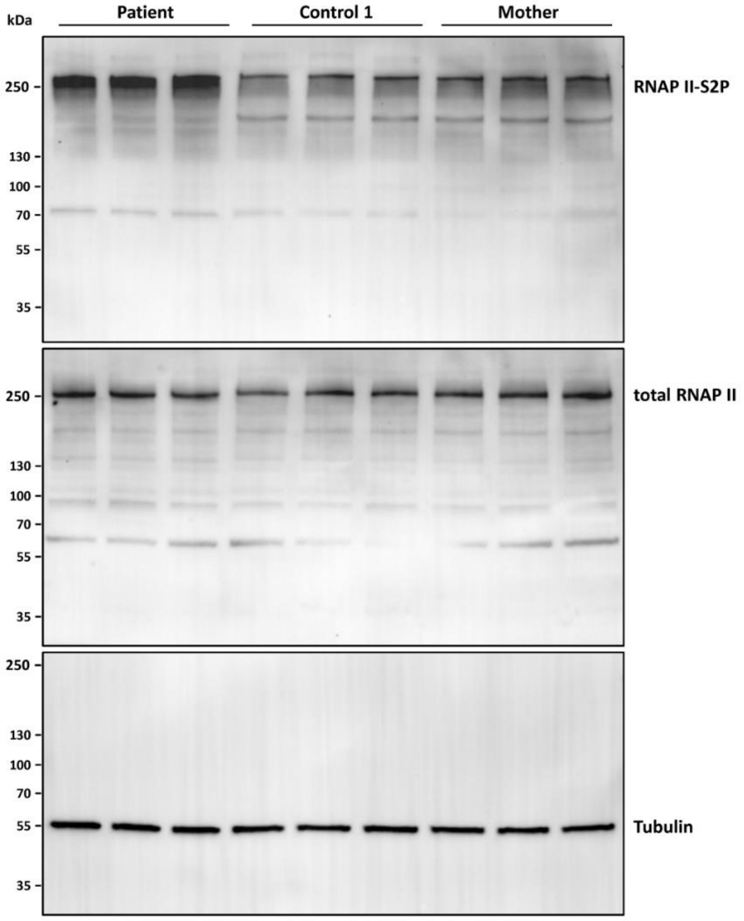

Figure S6. Full-length blots of Figure 4b

Immunoblots of lysates obtained from patient, control 1 and patient's mother fibroblast cultures from three different passages. The amount of total and phosphorylated RNAP II (RNAP II-S2P) was monitored with specific antibodies. An anti-Tubulin antibody was used to control for equal loading.

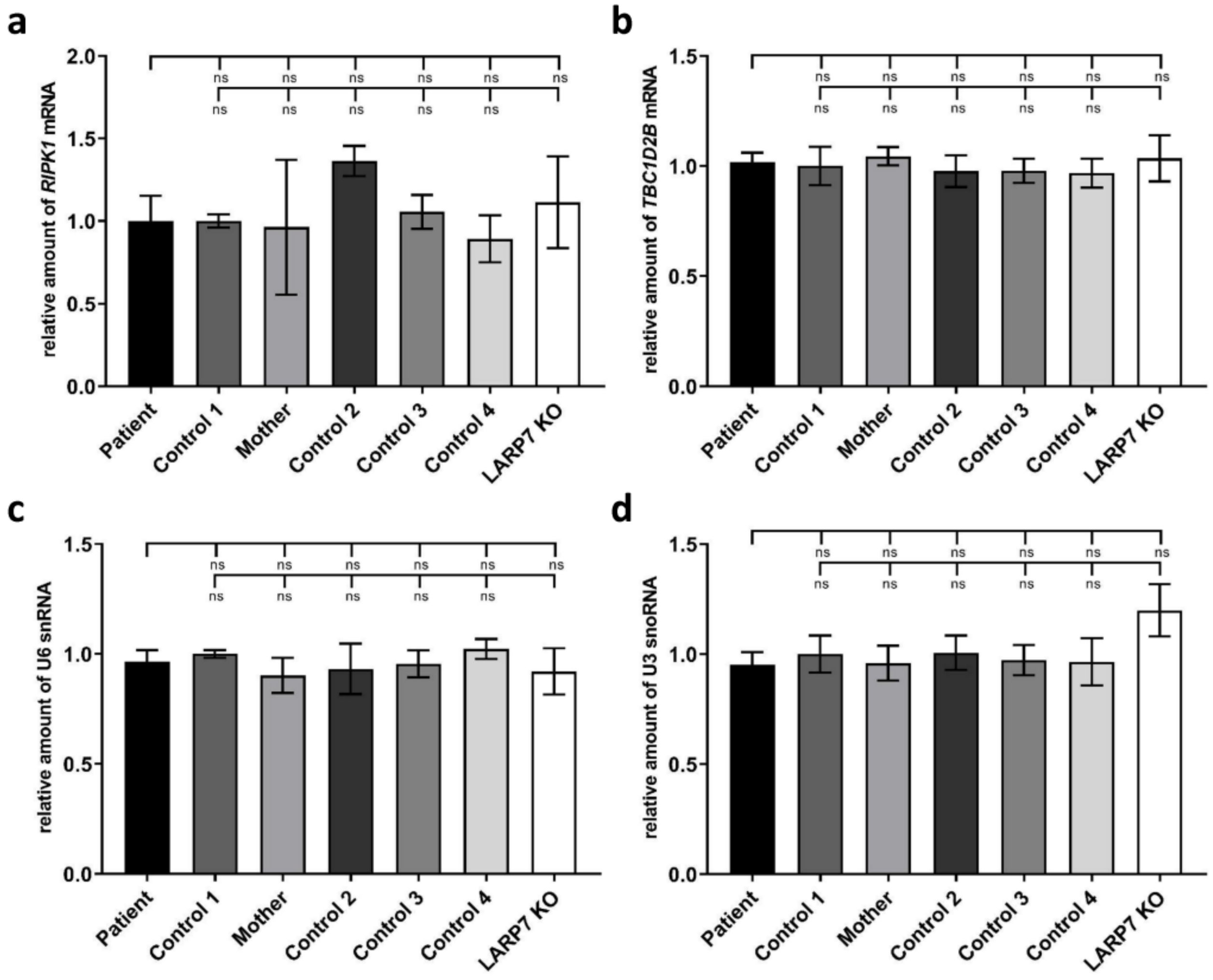

Figure S7. Expression of *RIKP1* and *TBC1D2B* mRNAs, U6 snRNA and U3 snoRNA is unchanged in patient-derived cells

Quantification of *RIPK1* **(a)** and *TBC1D2B* **(b)** mRNAs, U6 snRNA **(c)** and U3 snoRNA **(d)** by RT-qPCR. RNA was obtained from fibroblasts of the patient, the patient's mother, four healthy individuals (Controls 1-4) and LARP7 KO. *GAPDH* mRNA was used as an internal control, and the amount of each analysed RNA relative to *GAPDH* mRNA is presented. The mean of three independent experiments ± SD is given. A one-way ANOVA followed by Bonferroni *post-hoc* test for multiple comparison was performed. ns, not significant.

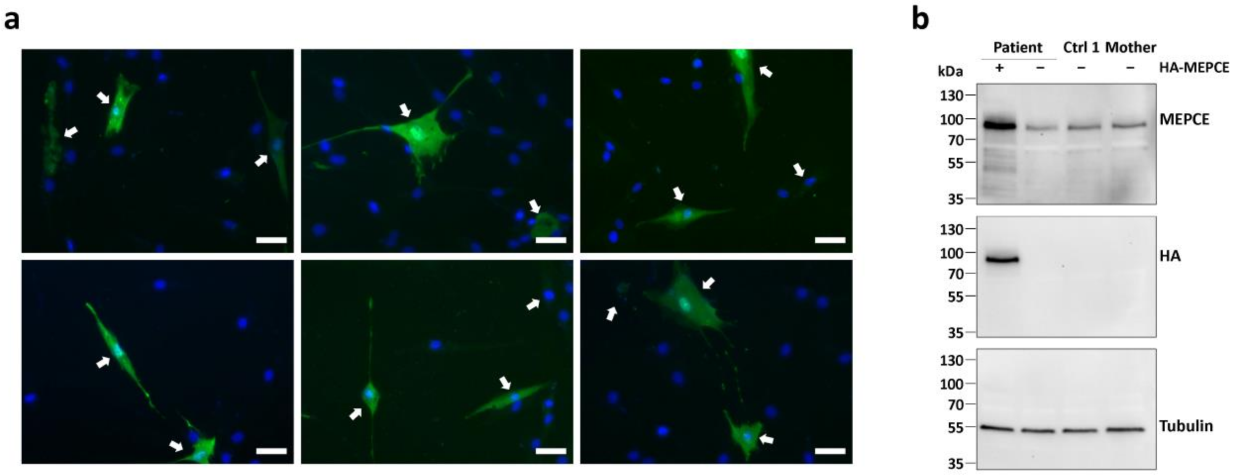

Figure S8. Transiently expressed HA-tagged MEPCE protein in patient fibroblasts detected by immunocytochemistry and immunoblotting

(a) Patient-derived fibroblasts were transiently transfected with HA-MEPCE expression construct, stained with mouse anti-HA antibody followed by anti-mouse Alexa Fluor 488-conjugated secondary antibody (green), and embedded in mounting solution with DAPI (blue). Six representative images are shown. Arrows point to transfected cells. We identified 40 cells expressing HA-MEPCE (green) by counting a total of 205 cells that yielded a transfection efficiency of 19.5%. Scale bar: 50 µm (white line). **(b)** Full-length immunoblots of lysates obtained from patient, control 1 and patient's mother fibroblasts. Fibroblasts of the patient were either transiently transfected with empty vector or HA-MEPCE expression construct (Patient + HA-MEPCE), while cells from a healthy individual (Ctrl 1) and the patient's mother were only transfected with HA-empty vector. The amount of total MEPCE protein was monitored with an anti-MEPCE antibody, the amount of overexpressed MEPCE protein was monitored with an anti-HA epitope-specific antibody. An anti-Tubulin antibody was used to control for equal loading. Representative blots are shown.

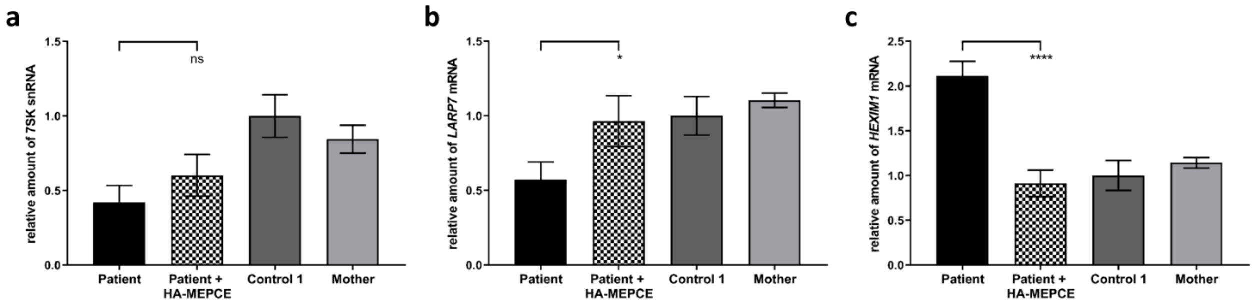

Figure S9. Expression of 7SK snRNA and *LARP7* and *HEXIM1* mRNAs after transient transfection of patient-derived fibroblasts with HA-MEPCE expression construct

Quantification of 7SK snRNA **(a)** and *LARP7* **(b)** and *HEXIM1* **(c)** transcripts by RT-qPCR. Fibroblasts of the patient were either transiently transfected with empty vector or HA-MEPCE expression construct (Patient + HA-MEPCE), while cells from a healthy individual (Control 1) and the patient's mother were only transfected with HA-empty vector. *GAPDH* mRNA was used as an internal control, and the amount of each analysed RNA relative to *GAPDH* mRNA is presented. The mean of three independent experiments ± SD is given. * $p \leq 0.05$; **** $p \leq 0.0001$ by one-way ANOVA followed by Bonferroni *post-hoc* test for multiple comparison. ns, not significant.

Chr.	Genomic position	Gene	mRNA reference number	Nucleotide change	Amino acid alteration	Zygosity	gnomAD browser: MAF [%]	CADD	REVEL	M-CAP	Splice site predictions	OMIM phenotype [MIM number]
7	100029193	*MEPCE*	NM_019606.6	c.1552C>T	p.(Arg518*)	*de novo*	Absent	35	NA	NA	Not done	NA
7	45015241	*MYO1G*	NM_033054.2	c.406G>A	p.(Asp136Asn)	*de novo*	Absent	13.99	0.076	0.008	No impact	NA
17	16852048	*TNFRSF13B*	NM_012452.2	c.445+4A>C	p.?	compound hetero-zygous	Absent	14.56	NA	NA	No impact	Immuno-deficiency, common variable, 2 [240500]; Immuno-globulin A deficiency 2 [609529]
	16875373			c.17G>A	p.(Arg6Gln)		0.004952	12.41	0.252	0.005	Not done	

Table S1. *In silico* pathogenicity and splice site predictions, minor allele frequency, and associated OMIM phenotypes for variants found in the patient
The functional impact of the identified variants was predicted by the Combined Annotation Dependent Depletion (CADD) tool, the Rare Exome Variant Ensemble Learner (REVEL) scoring system, and the Mendelian Clinically Applicable Pathogenicity (M-CAP) Score. CADD is a framework that integrates multiple annotations in one metric by contrasting variants that survived natural selection with simulated mutations. Reported CADD scores are phred-like rank scores based on the rank of that variant's score among all possible single nucleotide variants of hg19, with 10 corresponding to the top 10%, 20 at the top 1%, and 30 at the top 0.1%. The larger the score the more likely the variant has deleterious effects; the score range observed here is strongly supportive of pathogenicity, with all observed variants ranking above ~99% of all variants in a typical genome and scoring similarly to variants reported in ClinVar as pathogenic (~85% of which score >15) [1]. REVEL is an ensemble method predicting the pathogenicity of missense variants with a strength for distinguishing pathogenic from rare neutral variants with a score ranging from 0-1. The higher the score the more likely the variant is pathogenic [2]. M-CAP is a classifier for rare missense variants in the human genome, which combines previous pathogenicity scores (including SIFT, Polyphen-2, and CADD), amino acid conservation features and computed scores trained on mutations linked to Mendelian diseases. The recommended pathogenicity threshold is >0.025 [3]. Possible effects on splicing by the intronic *TNFRSF13B* variant and the *MYO1G* variant were analysed with the programs Human Splicing Finder 3.1, NetGene2 Server, and Berkeley Drosophila Genome Project Database [4, 5, 6, 7]. Chr., chromosome; MAF, minor allele frequency; NA, not applicable.

MEPCE primer sequences for variant validation		
**Template**	**Direction**	**Sequence (5' → 3')**
genomic DNA	Forward	GCCAAAACATCCGACACTAC
	Reverse	GCCTCTGGACCCTCTTAC
cDNA	Forward	GCCAAAACATCCGACACTAC
	Reverse	AGGCAGAGCACCACATCATA
**Primer sequences for RT-qPCR**		
**Gene**	**Direction**	**Sequence (5' → 3')**
MEPCE	Forward	TTGGATGGAGCGGACACATCAG
	Reverse	GAGGCAGAGCACCACATCATAC
LARP7	Forward	ACAGACAGTGGAGTACCTCAAAAC
	Reverse	CAGGTAGAGGCTCTGTGCTAATG
7SK snRNA	Forward	ATCTGTCACCCCATTGATCGCC
	Reverse	TCGTATACCCTTGACCGAAGAC
HEXIM1	Forward	CTTGTCAGAATATCAACACCAGCC
	Reverse	ATTGCCACCTACTGTCCTCCTC
ID2	Forward	TCGCATCCCACTATTGTCAGCC
	Reverse	AGCCACACAGTGCTTTGCTGTC
ID3	Forward	CTTAGCCAGGTGGAAATCCTACAG
	Reverse	GCTCCTTTTGTCGTTGGAGATG
MRPL11	Forward	AGGAGTTCAATGAGAGGACAAAG
	Reverse	CTTCAGGAAGTAGGAAACAGTGGG
U1 snRNA	Forward	ACTTACCTGGCAGGGGAGATAC
	Reverse	CCCACTACCACAAATTATGCAGTC
U2 snRNA	Forward	ATCGCTTCTCGGCCTTTTGG
	Reverse	GCTCCTATTCCATCTCCCTGCTC
U3 snoRNA	Forward	AGCACCGAAAACCACGAGGAAG
	Reverse	TCTCTCCCTCTCACTCCCCAATAC
U4 snRNA	Forward	GTATCGTAGCCAATGAGGTCTATCC
	Reverse	CCGTAGAGACTGTCAAAAATTGCC
U6 snRNA	Forward	CTCGCTTCGGCAGCACATATAC
	Reverse	TGGAACGCTTCACGAATTTGCG
RIPK1	Forward	CATGGAAAAGGCGTGATACACAAG
	Reverse	TTAAAGGAGGCAAGGCCGAG
TBC1D2B	Forward	TGCTCCAAGAAATGAAGACACCAG
	Reverse	CTTCCTCATCATCCTCAGGTACAG
GAPDH	Forward	TGACCCCTTCATTGACCTCAAC
	Reverse	GCATCGCCCCACTTGATTTTG

Table S2. Primer sequences used in this work

Supplemental references

1. Kircher, M. *et al.* A general framework for estimating the relative pathogenicity of human genetic variants. *Nat. Genet.* **46**, 310–315, doi:10.1038/ng.2892 (2014).
2. Ioannidis, N. M. *et al.* REVEL: An Ensemble Method for Predicting the Pathogenicity of Rare Missense Variants. *Am. J. Hum. Genet.* **99**, 877–885, doi:10.1016/j.ajhg.2016.08.016 (2016).
3. Jagadeesh, K. A. *et al.* M-CAP eliminates a majority of variants of uncertain significance in clinical exomes at high sensitivity. *Nat. Genet.* **48**, 1581–1586, doi:10.1038/ng.3703 (2016).
4. Brunak, S., Engelbrecht, J. & Knudsen, S. Prediction of human mRNA donor and acceptor sites from the DNA sequence. *J. Mol. Biol.* **220**, 49–65 (1991).
5. Desmet, F. O. *et al.* Human Splicing Finder: an online bioinformatics tool to predict splicing signals. *Nucleic Acids Res.* **37**, e67, doi:10.1093/nar/gkp215 (2009).
6. Hebsgaard, S. M. *et al.* Splice site prediction in Arabidopsis thaliana pre-mRNA by combining local and global sequence information. *Nucleic Acids Res.* **24**, 3439–3452 (1996).
7. Reese, M. G., Eeckman, F. H., Kulp, D. & Haussler, D. Improved splice site detection in Genie. *J. Comput. Biol.* **4**, 311–323, doi:10.1089/cmb.1997.4.311 (1997).
